# Plasma Exosomal MiRNAs Expression Profile in Mesial Temporal Lobe Epilepsy With Hippocampal Sclerosis: Case-Control Study and Analysis of Potential Functions

**DOI:** 10.3389/fnmol.2020.584828

**Published:** 2020-11-09

**Authors:** Li-Gang Huang, Yun-He Luo, Ji-Wen Xu, Qin-Chi Lu

**Affiliations:** ^1^Shanghai Sixth People’s Hospital, Shanghai Jiao Tong University, Shanghai, China; ^2^Minhang Hospital, Fudan University, Shanghai, China; ^3^School of Medicine, Renji Hospital, Shanghai Jiao Tong University, Shanghai, China

**Keywords:** exosome, miRNAs, mesial temporal lobe epilepsy, hippocampal sclerosis, stereo-electroencephalography

## Abstract

**Background:**

To explore an expression profile in plasma exosomal miRNAs of mesial temporal lobe epilepsy with hippocampal sclerosis (mTLE + HS) patients and investigate the associated clinical significance and putative pathways involved.

**Methods:**

Plasma exosomal miRNAs were measured in six mTLE + HS patients who were confirmed with pre-surgical stereo-electroencephalography and six without hippocampal sclerosis (mTLE−HS) using Illumina HiSeq 2500. Then six dysregulated miRNAs were chosen for validation in an independent sample of 18 mTLE + HS patients and 18 mTLE−HS controls using RT-qPCR. Receiver operating characteristic curve was conducted to evaluate the diagnostic value of miRNAs in HS. Bioinformatic analyses were conducted to reveal in which pathways these miRNAs were involved.

**Results:**

We revealed that a total of 42 exosomal miRNAs were differentially expressed in mTLE + HS. Among them, 25 were increased and 17 decreased. After validation, hsa-miR-129-5p, -214-3p, -219a-5p, and -34c-5p were confirmed as being upregulated, while hsa-miR-421 and -184 were significantly downregulated in mTLE + HS. Moreover, hsa-miR-184 had the best diagnostic value for discriminating mTLE + HS with 88.9% sensitivity and 83.3% specificity. These six miRNAs regulated several genes from neurotrophin-, hippo-, p53-, TGF- beta-, HIF- 1-, mTOR-related pathways.

**Conclusion:**

Six miRNAs were dysregulated in mTLE + HS patients and targeted several genes. This result might facilitate pathological mechanistic studies of miRNAs in HS and represent potential diagnostic biomarkers. These provided the rationale for further confirmation studies in larger cohorts of prospective patients.

## Key Points

1. Exosomal miRNA profile was detected in six mTLE + HS patients confirmed by SEEG.
2. Validation phase was performed in another 18 mTLE + HS patients.
3. Exosome-derived hsa-miR-184 might be as a significantly biomarker for mTLE + HS.

## Introduction

Mesial temporal lobe epilepsy with hippocampal sclerosis (mTLE + HS), which is the most common type of focal epilepsy, is clinically characterized by spontaneous recurrent seizures. Existing antiepileptic drugs (AEDs) are ineffective in approximately 30–35% of patients (Pitkanen et al., 2016). Furthermore, the long-term use of AEDs results in a heavy burden on society (Devinsky, 1999). Surgical removal of epileptogenic regions is an important treatment option for pharmacoresistant epilepsy (Ramey et al., 2013). However, epilepsy surgery is an invasive procedure with some risk of complications, including bleeding, infection, and stroke. In this context, identification of new biomarker for mTLE + HS may help accurately select these patients and, thus, may help monitor HS progression (Zhou, 2019).

Genome-wide miRNAs profiling studies based on hippocampal tissues (Kan et al., 2012; Roncon et al., 2015; Danis et al., 2016) and plasma (Wang et al., 2015a,b; Yan et al., 2017; Raoof et al., 2018) have identified various differentially expressed miRNAs. Using dual-center, dual-platform miRNA profiling, Raoof et al. (2018) have identified plasma miR-27a-3p, miR-328-3p, and miR-654-3p are potential biomarkers of TLE. Recent study on miRNA profiles of plasma exosomes from mTLE + HS patients compared with healthy controls has shown that hsa-miR-8071 has the best predictive value with a sensitivity of 83.33% and specificity of 96.67% (Yan et al., 2017). Therefore, exosomal miRNAs may be useful as biomarkers in mTLE + HS. However, the diagnosis of mTLE + HS performed in previous studies is mainly based on clinical examinations, including medical histories, cranial magnetic resonance imaging, ambulatory or video electroencephalogram (AEEG or VEEG). Hence, the exact discharge and severity of the pathologies associated with mTLE + HS are unclear in some cases, or the condition is even misdiagnosed.

Therefore, considering that miRNAs might be potential mTLE + HS biomarkers, we firstly investigated a circulating exosomal miRNA expression profile in the plasma of mTLE + HS patients which were confirmed by pre-surgical stereo-electroencephalography (SEEG) recordings, an accurate method for diagnosis (Li et al., 2016), aimed to find potential biomarker for accurately selecting these patients. We also carried out analyses to investigate the clinical significance and potential pathways in which it was involved.

## Materials and Methods

### Study Population

This multiphase case-control study was designed accordantly to STROBE guidelines, conducted to reveal differentially expressed plasma exosomal miRNAs and their potential roles for the early discrimination of mTLE + HS (von Elm et al., 2008; Supplementary Figure 1). This study included two independent samples of mTLE + HS patients and mTLE−HS controls, called the “Discovery phase” and the “Validation phase.” The “Discovery phase” included six mTLE + HS patients and six mTLE−HS controls. The “Validation phase” included 18 mTLE + HS patients and 18 mTLE−HS controls Table 1.

**TABLE 1:** Clinical characteristics of individuals.

Characteristics	Discovery phase		
	
	mTLE−HS (*n* = 6)	mTLE + HS (*n* = 6)	*p*-value
Gender (F:M)	3:3	4:2	0.557
Age (years)	22 (17.25–32)	29 (21.5–39.5)	0.493
Age at onset (years)	19 (11.5–27.25)	19.5 (7.75–26.75)	0.982
Aura (N:Y)	4:2	1:5	0.071
SE before surgery (N:Y)	6:0	5:1	1.000
IPI (N:Y)	3:3	4:2	0.557
Frequency (CPS per month)	3.5 (3–4.75)	10 (2.5–48.75)	0.281
Duration (years)	6.50 ± 7.12	12.17 ± 7.36	0.205
GTCS before surgery (N:Y)	5:1	1:5	**0.016**
Resection tissue (L:R)	2:4	1:5	0.502
FCD (I:II:III)	2:1:3	3:1:2	0.557
Follow up (month)	32.83 ± 5.85	35.67 ± 3.88	0.346
Engel (I:II:III:IV)	6:0:0:0	4:1:1:0	1.000

**Characteristics**	**Validation phase**		
	
	**mTLE−HS (*n* = 18)**	**mTLE + HS (*n* = 18)**	***p*-value**

Gender (F:M)	11:7	12:6	0.729
Age (years)	23 (20–38.75)	27 (22.75–31.75)	0.631
Age at onset (years)	11 (9–18.75)	9.5 (3.25–15.75)	0.227
Aura (N:Y)	8:10	9:9	0.738
SE before surgery (N:Y)	17:1	17:1	1.000
IPI (N:Y)	8:10	11:7	0.317
Frequency (CPS per month)	5 (3.25–25)	9 (2.25–27.5)	0.642
Duration (years)	12.44 ± 8.05	16.44 ± 8.20	0.149
GTCS before surgery (N:Y)	11:7	5:13	**0.044**
Resection tissue (L:R)	10:8	13:5	0.298
FCD (I:II:III)	7:1:10	10:1:7	0.317

*Quantitative variables with normal distribution were presented as mean ± SD, while skewed distribution were presented as median (25–75th percentiles). N, no; Y, yes; L, left; R, right; SD, standard deviation; SE, status epilepticus; IPI, initial precipitating incident; CPS, complex partial seizure; GTCS, generalized tonic clonic seizure; FCD, focal cortical dysplasia. Bold values represent that the incidence rate of GTCS before surgery was significantly higher between the mTLE patients with and without HS.*

All participants were recruited from the previous study (Huang et al., 2018). Patients in the “Discovery phase” were confirmed with pre-surgical SEEG recordings (Li et al., 2016) and pathologically diagnosed as mTLE + HS. Patients presenting recent episodes of chronic inflammatory diseases, stroke, chronic rheumatic disease, HIV-positivity, hepatitis, glucocorticoid treatment, smoking habits, alcoholism were excluded from the study. All participants had undergone anterior temporal lobectomy plus selective amygdala and hippocampus resection. The pathologies were referred to reference (Blumcke et al., 2011) and obtained from pathologic records. This study was approved by the Ethics Committee of Ren Ji Hospital, School of Medicine, Shanghai Jiao Tong University. All participating subjects provided written consent according to the Declaration of Helsinki.

### Plasma Samples

Peripheral blood samples from all subjects were collected in EDTA-coated tubes (6 ml/sample in Discovery phase, 2 ml/sample in Validation phase) before surgery. Plasma isolation was conducted within 3 h after collection by centrifugation for 5 min at 3,000 rpm at room temperature, followed by centrifugation for 5 min at 12,000 *g* at 4°C (Wang et al., 2015a). All samples were stored at −80°C and the hemolytic samples were excluded.

### Exosome Isolation

Exosomes were isolated from the plasma by QIAGEN exoRNeasy kit (QIAGEN GmbH, Hilden, Germany) according our previously works (Li et al., 2018; Wang et al., 2018). When the cold plasma was thawed at 4°C, it was subjected to successive centrifugations of 2,000 *g* for 20 min and 10,000 *g* for 20 min. Next, it was transferred into a new tube and placed at 4°C for 30 min with the mixed reagent. Exosomes pellets were centrifuged at 10,000 *g* for 30 min to remove the supernatant and were identified according the previously study (Li et al., 2018; Wang et al., 2018).

### Exosome Characterization

Ten microliters of resuspended plasma exosomes were loaded in 200 mesh carbon-coated copper grids for 1 min, then dried and negatively stained with 1% phosphotungstic acid for 20 s, blotted free of redundant liquid and dried under a lamp. Finally, the grids were detected with the H7650 transmission electron microscope (Hitachi, Japan).

### The Extraction and Quantity of Exosomal Total RNA

Exosomal total RNA was extracted via QIAGEN exoRNeasy kit (QIAGEN GmbH, Hilden, Germany) following the manufacturer’s procedure. The quantity of total RNA was monitored by Qubit (Thermo Fisher Scientific, Massachusetts, MA, United States). Only RNA samples that achieved adequate purity ratios (A260/A280 = 1.9–2.1) were used for analyses (Bustin et al., 2009). The integrity was analyzed via a Bioanalyzer 2100 and RNA 6000 Nano Lab Chip Kit (Agilent, California, CA, United States). The results of the Agilent 2200 Tape Station analysis of the 12 samples for sequencing were showed in Supplementary Figure 2.

### Small RNA Library Preparation and Sequencing

Approximately 50–120 ng of each total RNA were collected to prepare a small RNA library according to the manufacturer’s protocol for the TruSeq Small RNA Sample Prep Kit (Illumina, Inc., San Diego, CA, United States). Briefly, RNA molecules were sequentially ligated to 3′ and 5′ adaptors and then converted to cDNA by reverse transcription that was followed by polymerase chain reaction (PCR) amplification. The amplification products were excised from a 6% polyacrylamide Tris-Borate-EDTA gel. The purified cDNA library was used for cluster generation on a Cluster Station (Illumina, Inc.), and single-end sequencing was performed on a HiSeq 2500 (Illumina, Inc.) located at the RiboBio Co., Ltd. (Guangzhou, China) according to the manufacturer’s instructions. Raw sequencing reads were obtained with Sequencing Control Studio software (version 2.8; Illumina, Inc.) following the real-time sequencing image analysis and base-calling that were conducted with Real-Time Analysis software (version 1.8.70; Illumina, Inc.).

### Real Time-Quantitative PCR Measures the Expression of Specific miRNAs

Exosomal total RNA was extracted via QIAGEN exoRNeasy kit, then they were reverse-transcribed into cDNA using Prime Script TM RT-PCR Kit (Perfect Real Time) (RR037A, Takara, Dalian, China). RT-qPCR with SYBR^®^ Premix Ex Taq TM II (Tli RNaseH Plus) (RR820A, Takara, Dalian, China) was conducted with Light Cycler 480 (Roche, Germany) using the Bulge-Loop Primer Set (RiboBio, Guangzhou, China). In this study, miR-16-5p, one commonly used reference miRNA (Chen et al., 2005, 2008), was used as internal control of plasma miRNAs expression. The relative (fold-change) level of expression was analyzed by the comparative cycle threshold method (2^–Δ^ ^Δ^ ^*CT*^). The PCR primers were listed in Supplementary Table 1.

### MiRNA Target Genes Prediction and Pathway Analyses

Four target gene search tools [TargetScan (Lewis et al., 2005), miRDB (Wong and Wang, 2015), miRanda (Garcia et al., 2011), and CLIP-seq (Chi et al., 2009)] were used to find the list of target genes. Signaling pathways significantly enriched with target genes which were regulated by differentially expressed exosomal miRNAs were revealed by DIANA-mirPath v.3, which enabled false discovery rate correction (Benjamini and Hochberg) to the resulting significance levels (Vlachos et al., 2015). The TLE-associated miRNAs- targeted genes- signaling pathways network interactions were constructed and analyzed using MalaCards software, an integrated database of human disease (Rappaport et al., 2017).

### Statistics

Normal distribution was checked using the Kolmogorov–Smirnov and Shapiro–Wilk tests. Variables with normal distribution were presented as mean ± standard deviation (SD). Variables with skewed distribution were presented as median (25–75th percentiles). Clinical characteristics and miRNA expressions were compared using Student’s *t*-test or bilateral Chi-squared testing, as appropriate. To investigate the discriminatory power of the dysregulated miRNAs to distinguish between mTLE + HS patients and mTLE−HS subjects, receiver-operating characteristic (ROC) curve and the area under the ROC curve (AUC) were employed. Each experiment consisted of three replicates per condition. SPSS 17.0 software (IBM Corporation, Armonk, NY, United States) was used to perform all statistical analyses. *p-*values less than 0.05 were considered statistically significant. ^#^*p* < 0.05; ^∗^*p* < 0.01; ^∗∗^*p* < 0.001; ns, no significance.

## Results

### Clinical Characteristics of Participants

A total of 48 participants (including six mTLE + HS patients and six mTLE−HS controls in discovery phase, 18 mTLE + HS patients and 18 mTLE−HS controls in validation phase) were recruited to this study. The demographic and baseline characteristics were obtained from clinical or pathologic records. No significant differences in the gender, age, age at onset, frequency, duration or in the incidence rates of aura, status epilepticus before surgery and initial precipitating incident existed between the mTLE patients with and without HS. However, the incidence rate of GTCS before surgery was significantly higher between the mTLE patients with and without HS. The detailed demographic and clinical characteristics of individuals were listed in Supplementary Tables 2, 3.

### Distinct Profiles of Exosomal miRNAs of the mTLE−HS Controls vs. mTLE + HS Patients in the Discovery Phase

**FIGURE 1:**
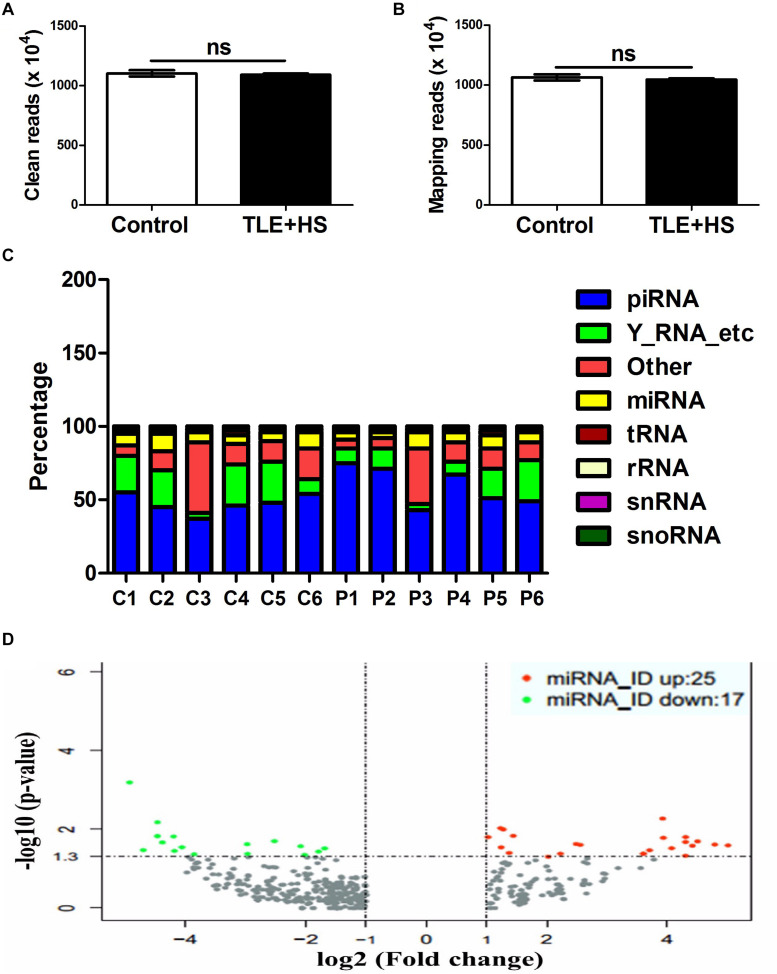
The circulating signatures of the exosome-derived miRNAs identified by HiSeq 2500 sequencing. The clean read numbers (**A**, *p* = 0.66) and mapping read numbers (**B**, *p* = 0.52) identified in the six mTLE–HS controls and six mTLE + HS patients. **(C)** The distribution of the mappable small RNAs detected by sequencing of the six mTLE–HS controls and six mTLE + HS samples. (piRNA, piwi-interacting RNA; Y-RNA-etc, Ro RNP; other, miscellaneous other RNA; miRNA, microRNA; tRNA, transfer RNA; rRNA, ribosomal RNA; snRNA, small nuclear RNA; snoRNA, small nucleolar RNA; P, patient; C, control.) **(D)** The volcano plot showing the relationship between the logarithm of the *p*-value on the *y*-axis and the log_2_ (fold-change) between the patients and controls on the *x*-axis. The vertical line marks the border between the results for the patients and those of the controls. Red indicates miRNAs with expression changes twofold greater than that of the remaining miRNAs, while green indicates miRNAs with expression changes less than 0.5-fold than that of the remaining miRNAs. (bar, SD.)

To comprehensively reveal differences in plasma exosomal miRNAs between the mTLE + HS and mTLE−HS groups, we carried out a RNAseq analysis. After removing the junk and low-quality reads, sequences shorter than 18 nucleotides, and adapter sequences, 11,031,025 and 10,903,495 clean reads on average remained in the six mTLE−HS controls and six mTLE + HS patients, respectively, for further analyses (Figure 1A). Among these clean reads, 10,649,494 (96.54%) and 10,465,566 (96.00%) average reads in the controls and patients, respectively, were mapped onto the human genome in GenBank (Figure 1B). The annotations of the percentage of RNA species were shown in Figure 1C. The miRNAs that were differentially expressed were identified using HiSeq 2500 sequencing. The genome-wide deep sequencing data and the analyses of the 513 exosomal miRNAs that were differentially expressed were listed in Supplementary Table 4. The miRNA level differences were considered statistically significant only if they met the following criteria (Wang et al., 2015a): (1) at least 10 copies in each group and (2) a fold-change (| log_2_ patient/control|) > 1 between the groups (*p* < 0.05). According to these criteria, we found that 8.2% (42/513) of the total miRNAs were differentially expressed (Supplementary Table 4, marked in yellow). Among them, there are 17 miRNAs (Supplementary Figure 3) were downregulated and 25 miRNAs (Supplementary Figure 4) were upregulated in mTLE + HS group (Figure 1D).

### Confirmation of Six Exosomal miRNAs Using RT-qPCR in the Validation Phase

**FIGURE 2:**
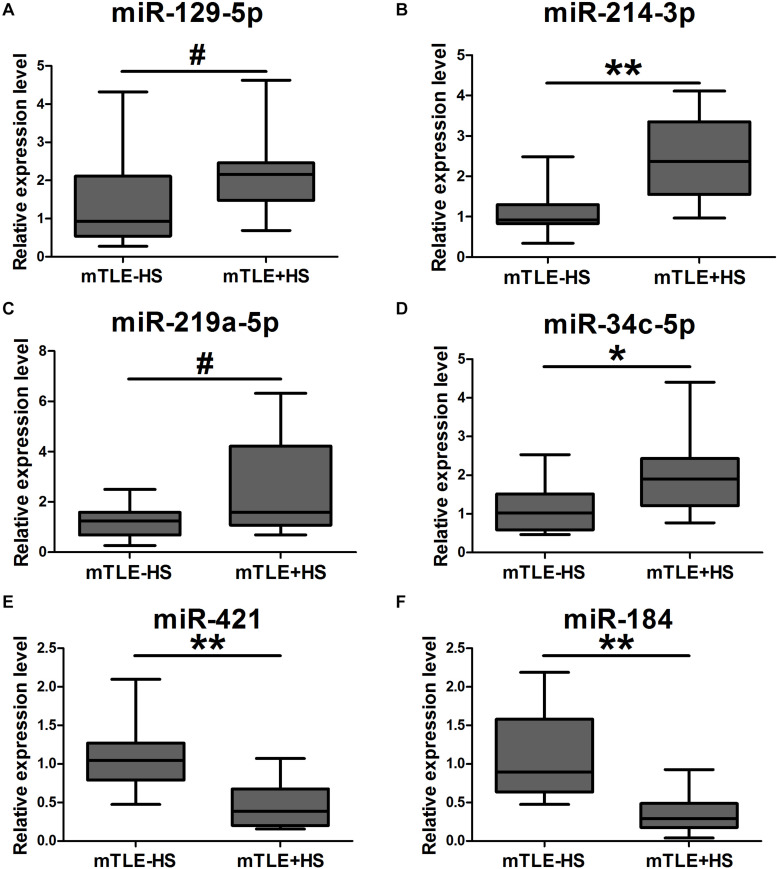
The expression of six exosomal miRNAs that were significantly dysregulated as compared between mTLE + HS patients and mTLE–HS controls. (**A**, *p* = 0.047; **B**, *p* = 1.57 × 10^– 5^; **C**, *p* = 0.014; **D**, *p* = 0.007; **E**, *p* = 4.10 × 10^– 5^; **F**, *p* = 6.95 × 10^– 6^; Box plot; All *n* = 18). ^#^*p* < 0.05; *p < 0.01; **p < 0.001.

Another 18 mTLE−HS controls and 18 mTLE + HS patients were recruited for the validation phase. The RT-qPCR analyses revealed that hsa-miR-129-5p [0.93 (0.58–1.97) vs. 2.16 (1.65–2.44)], miR-214-3p [0.92 (0.84–1.18) vs. 2.37 (1.70–3.24)], miR-219a-5p [1.24 (0.69–1.52) vs. 1.59 (1.09–3.58)], and miR-34c-5p [1.03 (0.61–1.34) vs. 1.91 (1.26–2.39)] were markedly upregulated, while miR-421 [1.05 (0.82–1.17) vs. 0.39 (0.21–0.60)] and miR-184 [0.90 (0.68–1.55) vs. 0.29 (0.19–0.45)] were significantly downregulated in the mTLE + HS group (Figure 2). Because the quality of RNA from the plasma samples was typically highly variable due to different degrees of RNA degradation, the mean Ct values for miRNAs of controls and patients were shown in Supplementary Table 5. A heat map of the high-throughput sequencing also showed that these six miRNAs were differentially expressed between the mTLE−HS control and mTLE + HS group (Supplementary Figure 5).

### Clinical Significance of Plasma Exosomal miRNAs as Potential Diagnostic Biomarker

**FIGURE 3:**
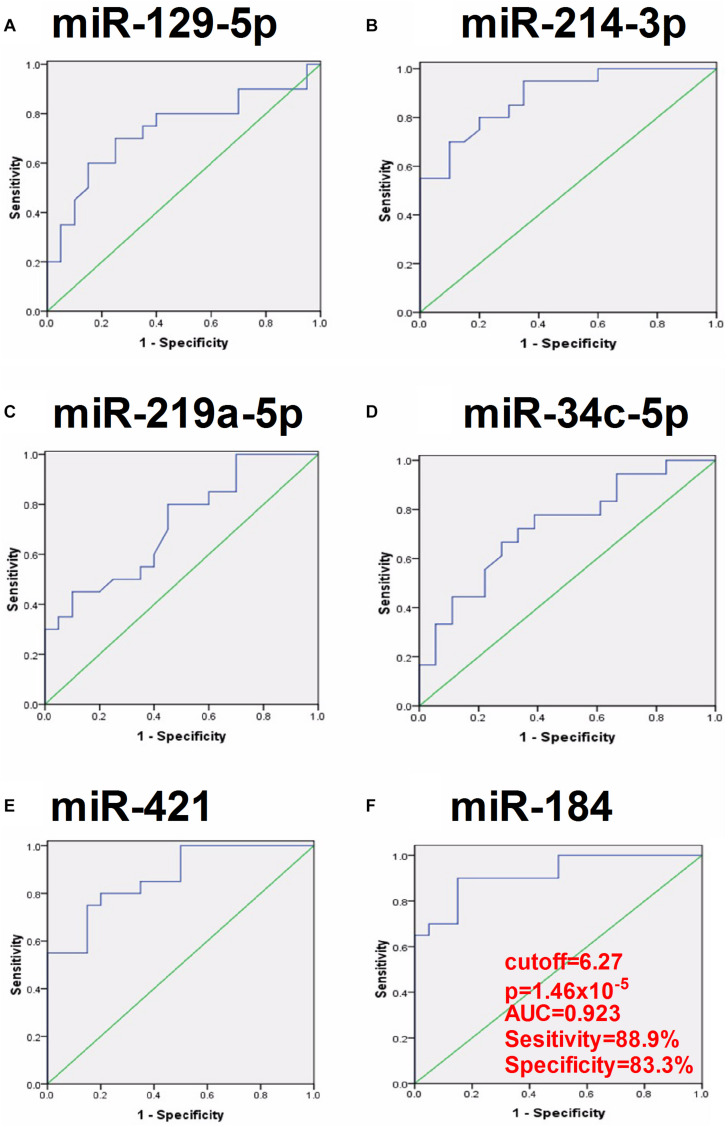
ROC curve analyses of the predictive values of the six exosomal miRNAs that were significantly dysregulated compared between the mTLE–HS controls and the mTLE + HS patients. (**A**, *p* = 0.016; **B**, *p* = 5.49 × 10^– 5^; **C**, *p* = 0.033; **D**, *p* = 0.007; **E**, *p* = 1.29 × 10^– 4^; **F**, *p* = 1.46 × 10^– 5^; AUC, area under the ROC curve).

To evaluate the clinical significance of plasma exosomal miRNA level in distinguishing mTLE + HS from mTLE−HS controls, ROC curve analysis was conducted. As shown in Figure 3 and Table 2, the AUC for hsa-miR-129-5p, -214-3p, -219a-5p, -34c-5p, -421, and -184 were found to be 0.735 (95% CI, 0.565–0.904), 0.894 (95% CI, 0.793–0.994), 0.708 (95% CI, 0.539–0.878), 0.764 (95% CI, 0.607–0.920), 0.873 (95% CI, 0.761–0.986), and 0.923 (95% CI, 0.819–1.000), respectively. Besides, the results revealed that hsa-miR-184 was the best marker for discriminating mTLE + HS from mTLE−HS control with an AUC = 0.923, and the best cutoff value of hsa-miR-184 was 6.265 with sensitivity and specificity of 88.9 and 83.3%, respectively, determined using Youden’s index. These results indicating that plasma exosomal hsa-miR-184 might predict the presence of mTLE + HS. In the 18 mTLE + HS patients, there were 6 men and 12 women with age ranging from 13 to 53 years. They were classified into a low hsa-miR-184 group (*n* = 9) and a high expression group (*n* = 9), according to the median level of all samples (median ΔCT value 7.38). We tested the association between its expression and clinical and pathological manifestations in order to better understand its potential role in the progression of mTLE + HS. We showed that the reduced abundance of hsa-miR-184 was likely correlated with an increased pre-surgical seizure frequency (Supplementary Table 6, *p* = 0.053). Altogether, these results suggested that exosomal hsa-miR-184 might play a role in the development and progression of mTLE + HS.

**TABLE 2:** The clinical significance of six plasma exosomal miRNA in distinguishing mTLE + HS from mTLE−HS controls.

Exosomal miRNA	Cutoff	p-Value	AUC	Sensitivity	Specificity	95% CI
hsa-miR-129-5p	5.34	0.016	0.735	0.722	0.722	0.565–0.904
hsa-miR-214-3p	5.28	5.49 × 10^–5^	0.894	0.833	0.778	0.793–0.994
hsa-miR-219a-5p	5.32	0.033	0.708	0.778	0.556	0.539–0.878
hsa-miR-34c-5p	5.94	0.007	0.764	0.667	0.778	0.607–0.920
hsa-miR-421	5.77	1.29 × 10^–4^	0.873	0.778	0.889	0.761–0.986
hsa-miR-184	6.27	1.46 × 10^–5^	0.923	0.889	0.833	0.819–1.000

### Prediction of miRNA Target Genes and Analysis of Pathway

Using four miRNA target genes search tools (TargetScan, miRDB, miRanda, and CLIP-seq), we found a list of potential target genes (*n* = 324, 148, 41, 64, 196, 14, respectively, Supplementary Table 7) predicted by all four software together in hsa-miR-129-5p, -214-3p, -219a-5p, -34c-5p, -421, and -184 (Supplementary Figure 6). Supplementary Table 8 showed the target genes pool with the target score >80 correlated to neuronal function using miRDB. We then conducted the DIANA-mirPath v.3 to identify potential affected pathways of aberrantly expressed miRNAs. By selecting TarBase v7.0 database and *p*-value threshold (0.05), this tool found 41 pathways potentially affected by hsa-miR-129-5p, -214-3p, -219a-5p, -34c-5p, -421, and -184 (Supplementary Table 9). The majority of affected target genes were correlated to molecular signaling pathways (hippo, p53, TGF-β, HIF-1, mTOR, neurotrophin, etc.) and cancer-related pathways (glioma, myeloid leukemia, prostate, melanoma, bladder, renal, colorectal, lung). Moreover, several pathways (Neurotrophin signaling pathways, Hippo signaling pathway, p53 signaling pathway, TGF-β signaling pathway, HIF-1 signaling pathway, mTOR signaling pathway), previously associated with neuronal function and epilepsy or brain development, were revealed as targets of specific miRNAs (*p* < 0.05; Supplementary Table 10).

### Construction of a TLE-Associated miRNA-Gene-Pathway Network

**FIGURE 4:**
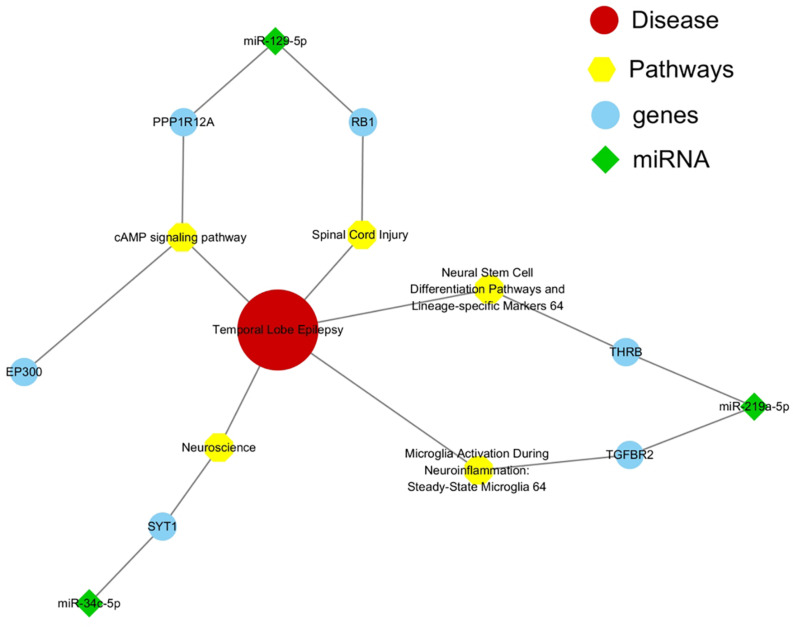
Interaction network among miRNAs and target genes and pathways. Green represented miRNAs, blue represented their target genes, and yellow represented signaling pathways which were significantly associated with mTLE + HS.

The functions of most miRNAs remained elusive and might be depended on the correlated protein-coding genes and specific signaling pathways. Therefore, we constructed and analyzed the network interactions among them using MalaCards software, an integrated database of human disease. By comparing all the genes of eight important pathways from MalaCards database with all the potential target genes of six miRNAs from Supplementary Table 7, we observed that only EP300 (-129-5p), PPP1R12A (-129-5p), RB1 (-129-5p), SYT1 (-34c-5p), TGFBR2 (-219a-5p), and THRB (-219a-5p) were associated with mTLE + HS (Figure 4).

## Discussion

Surgery is an effective procedure for mTLE + HS patients. We aim to find potential biomarker for accurately selecting these patients. mTLE + HS used in previous studies was mainly based on AEEG or VEEG recordings, sometimes the condition was inevitable misdiagnosed. Here we firstly selected mTLE + HS patients with a diagnosis confirmed by pre-surgical SEEG recordings. We observed that 8.2% of the total miRNAs were differentially expressed, which was in agreement with the existing literature (Wang et al., 2015a). After the validation, there were six exosomal miRNAs which were markedly dysregulated. In particular, hsa-miR-184 showed the best predictive value for mTLE + HS. Therefore, we believed that our study provided high-quality evidence.

Notably, previously miRNA expression profiling studies had shown that plasma hsa-miR-184 was increased in epilepsy patients (Wang et al., 2015b), but it was decreased in patients with drug-resistant epilepsy (Wang et al., 2015a). In addition, hsa-miR-184 level in hippocampal tissues of TLE + HS patients was identified as greatly increased in Kaalund’s study (Kaalund et al., 2014), while it was significantly decreased in Kan’s and Danis’s study (Kan et al., 2012; Danis et al., 2016). These discrepancies might be due to the different criteria used to select patients for surgery and used to identify significant dysregulations of miRNAs. Moreover, factors such as different models and brain regions, limited sample sizes, and extraneous aspects including race and lifestyle, might also affect the profiles of miRNA expression. Therefore, additional large-scale studies were still needed in the future to verify this result.

Danis’s study showed that genome miRNA sequencing in hippocampus tissues did not reveal obvious differences between mTLE patients with and without HS, but only hsa-miR-184 was significantly decreased confirmed by further RT-qPCR. Whether detected hsa-miR-184 was secreted from the hippocampus or other brain regions exactly remained unclear (Danis et al., 2016). In our study, the reduced abundance of plasma exosomal hsa-miR-184 was likely correlated with an increased pre-surgical seizure frequency, but it remarkably increased to detectable range 1 week after operation (Supplementary Figure 7), suggesting that hsa-miR-184 would be derived from anterior temporal lobe, amygdala or hippocampus. Foldvary et al. (2000) had reported that seizure frequency was divided in 1–5, 6–10, 11–20, and more than 20 seizures per month. In our cases, the frequency (CPS per month) had been roughly dichotomized into <5 and ≥5 because the number of patients was limited. We only detect expression of hsa-miR-184 in one point after operation, it might keep rising and manage to reach the summit of level, then contribute to prevent epileptogenesis and ictogenesis (Cattani et al., 2016).

There have been some reports of the targets and functions related to hsa-miR-184. AKT2, BIN3, and PRKCB, genes regulated by miR-184, were known to be involved in immune response, inflammatory and apoptosis related pathways (Danis et al., 2016). McKiernan’s study showed that inhibiting miR-184 *in vivo* resulted in neuronal death after preconditioning seizures and aggravated seizure-induced neuronal death following status epilepticus, which suggested that miR-184 was a novel contributor to neuronal survival following mild and severe seizures (McKiernan et al., 2012). However, it remained unclear where exosomal miR-184 was produced, in which tissues and cells it functions, and how it was involved in epileptogenesis.

Previous profiling and target studies had also reported an upregulated level of hsa-miR-129-5p, which were consistent with the result of our study (Kan et al., 2012; Sosanya et al., 2015). However, Wang’s study reported that serum hsa-miR-129-5p was downregulated in epilepsy patients (Wang et al., 2015b). As to miR-219a-5p, a previous study had reported that it was decreased in both kainic acid (KA)-induced animal model and in the cerebrospinal fluid samples of epilepsy patients (Zheng et al., 2016). For miR-34c-5p, it was markedly over-expressed in a cortical tuberous sclerosis complex (Mills et al., 2017), which was associated with intellectual disability, autism, and severe epilepsy (Dombkowski et al., 2016). As to miR-421, previous studies had reported that it was increased in the plasma of drug-resistant epilepsy patients (Wang et al., 2015a).

The mammalian target of rapamycin (m-TOR), which was a serine/threonine protein kinase that regulated activity-dependent protein synthesis in neurons, was overactive in epilepsy, suggesting that excessive protein synthesis might contribute to the neuronal pathology of epilepsy. The upregulated rno-miR-129 might result in a reduction of the voltage-gated potassium channel Kv1.1 when mTORC1 was active in TLE models (Sosanya et al., 2013; Sosanya et al., 2015). N-methyl-D-aspartate (NMDA) receptors played an important role in epileptogenesis and NMDA receptor antagonists had been found to have antiepileptic effects in humans and animals (Rothan et al., 2017). The miR-219a-5p was a brain-specific miRNA and had been shown to negatively regulate the function of NMDA receptors by targeting Ca^2+^/calmodul independent protein kinase II (CaMKII)γ (Kocerha et al., 2009). Because miR-219a-5p up-regulation induced downregulation of CaMKIIγ in KA-treated mice, this might be a compensatory mechanism in epilepsy. Thus, delivery of miR-219a-5p might represent an anti-epileptogenic therapeutic strategy (Zheng et al., 2016). Interestingly, recent studies had revealed that a higher level of the miR-34 family (a/b/c) helped in maintaining neuronal differentiation by arresting cells in the G1 phase (Jauhari et al., 2018) and contributed to individual responsiveness to stress by affecting the 5-HT prefrontal/γ-aminobutyric acid amygdalar system (Andolina et al., 2018).

A subset of four miRNAs (miR-155-5p, miR-146a-5p, miR-134-5p, and miR-218-5p), which were shown to be without aberrant regulation in the present study, were previously identified as showing differential expression in epilepsy. This discrepancy might be due to differences in the studied organism, sample type, technical approach, and epilepsy subtype explored in different studies. For miR-155-5p, Wang et al. (2015a) showed it was up-regulated in drug-resistant epilepsy. It had also been found dysregulated in stroke (Caballero-Garrido et al., 2015) and glioma (Wu et al., 2017). miR-155-5p silencing after ischemia might play a role improving recovery (Caballero-Garrido et al., 2015). Glioma, stroke, and epilepsy were all associated with neural diseases, thus, it was possible that miR-155-5p might participate in the pathogenesis of these three diseases through common pathways. With respect to miR-146a-5p, many studies had shown that it was increased in both epileptic humans and rats (Roncon et al., 2015; Wang et al., 2015a). van Spronsen et al. (2013) demonstrated that miR-146a-5p showed altered patterns of expression after NMDA receptor-dependent plasticity. Because miR-146a-5p up-regulation induced downregulation of pro-inflammatory cytokines, it might be a compensatory mechanism (Iyer et al., 2012). With respect to miR-134-5p, Wang et al. (2017) showed it was up-regulated in new-onset epilepsy patients. Interestingly, inhibiting miR-134-5p expression could suppress prolonged seizures and result in neuroprotection in multiple models of epilepsy (Jimenez-Mateos et al., 2012). As for miR-218-5p, previous studies had reported that it was decreased in mTLE + HS patients and affected axonal guidance as well as synaptic plasticity by regulating *SLC1A2* expression (Kaalund et al., 2014).

Because the gene expression and pathways depended partly on the balance of miRNAs, an abnormal dysregulation of specific miRNA might eventually result in disease pathology. We employed bioinformatic prediction tools (TargetScan, miRDB, miRanda, and CLIP-seq) and analysis software (DIANA-mirPath and MalaCards), to explore a list of predicted target genes and enriched pathways.

All four tools identified a series of potential target genes together affected by altered expression miRNAs newly discovered in mTLE + HS patients. Especially, CDKN1C (-129-5p), ATP2B4 (-129-5p, -214-3p), KIAA1217 (-34c-5p), ELAVL2 (-421), and SPRY1 (-421) gene expression were differential in dentate granule cells of hippocampus tissues in mTLE + HS patients (Griffin et al., 2016). Four of these six miRNAs potentially regulate the gene expression of potassium voltage-gated channel subfamily members and gamma-aminobutyric acid (GABA) pathway, widely prevailing epilepsy origin and ictogenesis hypothesis (DiNuzzo et al., 2014). DIANA-mirPath software had also generated several significantly pathways (cancer, signaling, etc.) potentially affected by altered expressed miRNAs (Supplementary Table 9). Supplementary Table 10 contained a subset of KEGG pathways previously related to epilepsy.

*P53* signaling pathway represented an important stage in the formation of inflammation and oxidative stress in various factors (e.g., ischemia, hypoxia), affecting the internal environment homeostasis caused by seizures. In our previous study, we reported that a knock-down of miR-155-5p might inhibit epileptogenesis by activating *SESN3*, a protein code gene of *p53* signaling pathway downstream. Moreover, inhibition of *SESN3* function might induce related gene expression and then aggravate epileptogenesis (Huang et al., 2018). Another was neurotrophin signaling pathway, which might affect neural excitability through controlling the differentiation, remodeling, and survival of neurons (Xu et al., 2002). Furthermore, we constructed and analyzed the miRNA-mRNA-pathway interaction network based on ceRNAs using MalaCards software to reveal the role of these six miRNAs in the pathological process of mTLE + HS. It showed that EP300, PPP1R12A, RB1, SYT1, TGFBR2, and THRB were associated with mTLE + HS. The present results suggested that plasma exosomal hsa-miR-184 might predict the presence of mTLE + HS. However, for significant potential target genes and enriched pathway associated with mTLE + HS were related to miR-129-5p, miR-219a-5p and miR-34c-5p instead of miR-184. It partly because the limited sample size in the validation phase resulting in inaccurate result. However, these genes needed to be confirmed via functional experiments on epilepsy models or cell cultures.

Although this study was not the first to analyze the expression profiles of exosomal miRNAs in the context of epilepsy, one implication of this study was that the genome-wide exosome-derived miRNA expression was profiled in mTLE + HS patients confirmed by SEEG recordings. However, this study had several limitations. First, the sample size in the validation phase was not much larger than those of previous studies, and it should be at least 30 according to clinical epidemiology research center of the Third Hospital of Peking University. Clinical factors and other important confounders such as age at onset and proportion of gender could affect the level of miRNAs. The plasma samples were isolated within 3 h after collection, it was possible that some low expressed miRNAs could be lost during the extraction process. Second, this was a single-center work, additional samples from multi-center studies, however, were needed to verify this finding. Third, although we had analyzed 42 plasma exosomal miRNAs which were differentially expressed by sequencing, we could not exclude the possibility that other important mTLE + HS correlated miRNAs were not analyzed, and the direct and indirect functions of these six miRNAs must be investigated in the future. This finding must be validated by multivariate logistic regression analyses on multiple variables, and conducted in additional larger-scale prospective studies in different ethnic populations.

## Conclusion

In conclusion, the present study identified a set of 42 dysregulated miRNAs in mTLE + HS patients with diagnosis by SEEG. Six of these were validated in an independent sample, confirming that hsa-miR-129-5p, -214-3p, -219a-5p, and -34c-5p were upregulated, miR-421 and -184 were downregulated in mTLE + HS patients. The most interesting finding of this study was that hsa-miR-184 might be as a potential biomarker for mTLE + HS, which required further investigation in different populations, as well as evaluation of its function on target genes and putative pathways.

## Data Availability Statement

The datasets analyzed in this study. This data can be found here: https://www.ncbi.nlm.nih.gov/sra/?term=SRP273213.

## Ethics Statement

The studies involving human participants were reviewed and approved by Ethics Committee of Ren Ji Hospital, School of Medicine, Shanghai Jiao Tong University. Written informed consent to participate in this study was provided by the participants’ legal guardian/next of kin. Written informed consent was obtained from the individual(s), and minor(s)’ legal guardian/next of kin, for the publication of any potentially identifiable images or data included in this article.

## Author Contributions

Q-CL conceived the project. J-WX participated in the discussion and proposed helpful suggestions. L-GH and Y-HL designed the experiments, carried out the majority of the experiments and wrote the manuscript. All authors contributed to the article and approved the submitted version.

## Conflict of Interest

The authors declare that the research was conducted in the absence of any commercial or financial relationships that could be construed as a potential conflict of interest.
