# Supplementary material for: Plasma Exosomal MiRNAs Expression Profile in Mesial Temporal Lobe Epilepsy With Hippocampal Sclerosis: Case-Control Study and Analysis of Potential Functions
Source: Front Mol Neurosci. 2020 Nov 9;13:584828. doi: 10.3389/fnmol.2020.584828 (PMC7680973; doi:10.3389/fnmol.2020.584828)
Supplement: Supplementary file 1 [file Data_Sheet_1.ZIP › 0718Supplementary Material.doc]

**Plasma Exosomal MiRNAs Expression Profile in Mesial Temporal Lobe Epilepsy with Hippocampal Sclerosis: Case-control Study and Analysis of Potential Functions**

Li-Gang Huang^#1^^,2^, Yun-He Luo^#2^, Ji-Wen Xu^3^, Qin-Chi Lu*^4^

^1^Department of Neurology, Shanghai Jiao Tong University Affiliated Sixth People's Hospital, Shanghai, China;

^2^Department of Neurology, Min Hang Hospital, Fu Dan University, Shanghai, China;

^3^Department of Functional Neurosurgery, Ren Ji Hospital, School of Medicine, Shanghai Jiao Tong University, Shanghai, China;

^4^Department of Neurology, Ren Ji Hospital, School of Medicine, Shanghai Jiao Tong University, Shanghai, China.

**^#^**Equal contributors

**Corresponding author**:

Name: Qin-Chi Lu, MD, PhD.

E-mail: longmanmei1989@163.com

**Figure S1. (page 3)**

**Figure S2. (page 4-18)**

**Figure S3. (page 19)**

**Figure S4. (page 20)**

**Figure S5. (page 21)**

**Figure S6. (page 22-27)**

**Figure S7. (page 28)**

**Table S1. (page 29-30)**

**Table S2. (page 31-32)**

**Table S3. (page 33-36)**

**Table S4. (page 37-56)**

**Table S5. (page 57-59)**

**Table S6. (page 60-61)**

**Table S7. (page 62-74)**

**Table S8. (page 75-76)**

**Table S9. (page 77-78)**

**Table S10. (page 79-80)**

**Figure S1** Flowchart of the study design.

**Figure S1** Discovery phase: microarray analysis using Illumina HiSeq 2500. miRNAs have at least 10 copies in mTLE+HS and mTLE-HS control and show a fold-change (∣log_2_ patient/control∣) > 1 between the groups (*p* < 0.05). Validation phase: RT-qPCR analyses on individual samples. miRNAs have a Ct value < 36 and detection rate > 75% (*p* < 0.05). (P, patient; C, control; ROC, receiver-operating characteristic).

**Figure S2**

**Agilent 2200 Tape Station analysis. (**P, patient; C, control).

| Sample | Qubit. Con. (ng/μl) | Mass (μg) |
| --- | --- | --- |
| Patient 1 | 2.200 | 0.066 |
| Patient 2 | 2.200 | 0.055 |
| Patient 3 | 2.000 | 0.050 |
| Patient 4 | 2.800 | 0.084 |
| Patient 5 | 2.100 | 0.074 |
| Patient 6 | 2.000 | 0.060 |
| Control 1 | 2.200 | 0.055 |
| Control 2 | 3.990 | 0.120 |
| Control 3 | 3.570 | 0.107 |
| Control 4 | 3.770 | 0.113 |
| Control 5 | 2.510 | 0.075 |
| Control 6 | 2.000 | 0.060 |
|  | | |

**Location 0. Sample name：**High sensitivity RNA ladder

**Location 1. Sample name：patient 1**

**Location 3. Sample name：patient 2**

**Location 4. Sample name：patient 3**

**Location 6. Sample name：patient 4**

**Location 8. Sample name：patient 5**

**Location 9. Sample name：patient 6**

**Location 10. Sample name：control 1**

**Location 11. Sample name：control 2**

**Location 12. Sample name：control 3**

**Location 13. Sample name：control 4**

**Location 16. Sample name：control 5**

**Location 18. Sample name：control 6**

**Figure S3** Differentially downregulated 17 exosomal miRNAs.

**Figure S4** Differentially upregulated 25 exosomal miRNAs.

**Figure S5** Heat map of the results of the high-throughput sequencing showed the differential level of exosome-derived miRNA expression between the 6 mTLE-HS controls and 6 mTLE+HS patients. (P, patient; C, control; Blue, decrease; Red, increase).

**Figure S6** Venn diagram showed the potential miRNA target genes predicted by all four software.

A. hsa-miR-129-5p

B. hsa-miR-214-3p

C. hsa-miR-219a-5p

D. hsa-miR-34c-5p

E. hsa-miR-421

F. hsa-miR-184

**Figure S7** Plasma exosomal hsa-miR-184 level was increased 1 week after operation in 18 mTLE+HS patients.

**Table S1 The primer sequences used for RT-qPCR.**

| Genes (Homo sapiens) | Primers | Sequences(5′- 3′) |
| --- | --- | --- |
| miR-129-5p | RT primer | GTCGTATCCAGTGCAGGGTCCGAGG |
|  |  | TATTCGCACTGGATACGACGCAAGC |
|  | Forward | GCGCGCTTTTTGCGGTCTGG |
|  | Reverse | ATCCAGTGCAGGGTCCGAGG |
|  |  |  |
| miR-214-3p | RT primer | GTCGTATCCAGTGCAGGGTCCGAGG |
|  |  | TATTCGCACTGGATACGACACTGCC |
|  | Forward | CGCGACAGCAGGCACAGACA |
|  | Reverse | ATCCAGTGCAGGGTCCGAGG |
|  |  |  |
| miR-219a-5p | RT primer | GTCGTATCCAGTGCAGGGTCCGAGG |
|  |  | TATTCGCACTGGATACGACAGAATT |
|  | Forward | CGCGCGTGATTGTCCAAACGC |
|  | Reverse | ATCCAGTGCAGGGTCCGAGG |
|  |  |  |
| miR-34c-5p | RT primer | GTCGTATCCAGTGCAGGGTCCGAGG |
|  |  | TATTCGCACTGGATACGACGCAATC |
|  | Forward | CGCGAGGCAGTGTAGTTAGCT |
|  | Reverse | AGTGCAGGGTCCGAGGTATT |
|  |  |  |
| miR-421 | RT primer | GTCGTATCCAGTGCAGGGTCCGAGG |
|  |  | TATTCGCACTGGATACGACGCGCCC |
|  | Forward | GCGCGATCAACAGACATTAATT |
|  | Reverse | AGTGCAGGGTCCGAGGTATT |
|  |  |  |
| miR-184 | RT primer | GTCGTATCCAGTGCAGGGTCCGAGGT |
|  |  | ATTCGCACTGGATACGACACCCTT |
|  | Forward | GCGCGTGGACGGAGAACTGAT |
|  | Reverse | ATCCAGTGCAGGGTCCGAGG |
|  |  |  |
| miR-16-5p | RT primer | GTCGTATCCAGTGCAGGGTCCGAGG |
|  |  | TATTCGCACTGGATACGACCGCCAA |
|  | Forward | TCGGCGGTAGCAGCACGTAAATA |
|  | Reverse | ATCCAGTGCAGGGTCCGAGG |

**Table S2** Demographics and clinical features of 6 mTLE-HS controls and 6 mTLE+HS patients for sequencing.

| Num. | Gender | Age (yr) | Age at onset (yr) | Aura | SE before surgery | IPI | Frequency (CPS per month) | Duration (yr) |
| --- | --- | --- | --- | --- | --- | --- | --- | --- |
| 1 TLE-HS | M | 49 | 29 | N | N | N | 1 | 20 |
| 2 TLE-HS | F | 15 | 10 | N | N | Y | 3 | 5 |
| 3 TLE-HS | M | 34 | 33 | N | N | Y | 3 | 1 |
| 4 TLE-HS | F | 18 | 10 | N | N | N | 5 | 8 |
| 5 TLE-HS | M | 17 | 16 | Y | N | N | 4 | 1 |
| 6 TLE-HS | F | 26 | 22 | Y | N | Y | 10 | 4 |
| 1 TLE+HS | F | 20 | 7 | N | N | Y | 12 | 13 |
| 2 TLE+HS | M | 32 | 8 | Y | N | Y | 1 | 24 |
| 3 TLE+HS | M | 62 | 47 | Y | Y | N | 8 | 15 |
| 4 TLE+HS | F | 28 | 20 | Y | N | N | 15 | 8 |
| 5 TLE+HS | F | 22 | 20 | Y | N | N | 3 | 2 |
| 6 TLE+HS | F | 30 | 19 | Y | N | N | 150 | 11 |
| Continued |  |  |  |  |  |  |  |  |
| Num. | GTCS before surgery | AEDs before surgery | SEEG | HS | Resection tissue | FCD | Follow up (month) | Engel |
| 1 TLE-HS | Y | LEV,VPA,OXC | N | N | R | II | 27 | I |
| 2 TLE-HS | N | CBZ,LEV，TPM | N | N | R | III | 34 | I |
| 3 TLE-HS | N | CBZ,VPA,GBP | N | N | L | III | 38 | I |
| 4 TLE-HS | N | VPA,LEV | Y | N | R | I | 41 | I |
| 5 TLE-HS | N | VPA,CBZ | Y | N | L | I | 30 | I |
| 6 TLE-HS | N | LEV,TPM,CBZ,OXC,LTG | Y | N | R | III | 27 | I |
| 1 TLE+HS | Y | PB,CBZ,VPA,LTG,OXC | Y | Y | L | III | 33 | I |
| 2 TLE+HS | N | VPA,OXC | Y | Y | R | III | 40 | I |
| 3 TLE+HS | Y | VPA,LTG | Y | Y | R | II | 41 | I |
| 4 TLE+HS | Y | LEV,LTG | Y | Y | R | I | 33 | II |
| 5 TLE+HS | Y | OXC,LEV | Y | Y | R | I | 32 | I |
| 6 TLE+HS | Y | OXC,VPA | Y | Y | R | I | 35 | III |

F, female; M, male; Y, yes; N, no; L, left; R, right; IPI, initial precipitating incident (e.g., trauma, ischemic injury, encephalitis); CPS, complex partial seizure; GTCS, generalized tonic clonic seizure; SE, status epilepticus; HS, hippocampal sclerosis; AEDs, antiepileptic drugs; VPA, valproic acid; CBZ, carbamazepine; LTG, lamotrigine; OXC, oxcarbazepine; LEV, levetiracetam; PB, phenobarbital; SEEG, stereo-electroencephalography; FCD, focal cortical dysplasia.

**Table S3 Demographics and clinical features of 18 mTLE-HS controls and 18 mTLE+HS patients for validation phase.**

| Num. | Gender | Age (yr) | Age at onset (yr) | Aura | SE before surgery | IPI | Frequency (CPS per month) |
| --- | --- | --- | --- | --- | --- | --- | --- |
| 1 TLE-HS | M | 23 | 7 | Y | N | N | 4 |
| 2 TLE-HS | F | 19 | 10 | Y | Y | Y | 3 |
| 3 TLE-HS | F | 21 | 15 | N | N | Y | 10 |
| 4 TLE-HS | F | 38 | 20 | Y | N | N | 1 |
| 5 TLE-HS | F | 50 | 40 | N | N | N | 1 |
| 6 TLE-HS | F | 16 | 11 | N | N | Y | 5 |
| 7 TLE-HS | F | 42 | 7 | N | N | Y | 10 |
| 8 TLE-HS | F | 24 | 11 | Y | N | N | 5 |
| 9 TLE-HS | M | 23 | 15 | Y | N | N | 4 |
| 10 TLE-HS | M | 20 | 1 | N | N | Y | 3 |
| 11 TLE-HS | M | 32 | 9 | Y | N | Y | 10 |
| 12 TLE-HS | F | 48 | 44 | N | N | N | 30 |
| 13 TLE-HS | M | 20 | 15 | Y | N | N | 60 |
| 14 TLE-HS | M | 69 | 67 | N | N | Y | 60 |
| 15 TLE-HS | F | 21 | 9 | Y | N | Y | 5 |
| 16 TLE-HS | F | 20 | 5 | Y | N | Y | 2 |
| 17 TLE-HS | M | 39 | 24 | Y | N | N | 60 |
| 18 TLE-HS | F | 20 | 11 | N | N | Y | 60 |
| 1 TLE+HS | F | 13 | 2 | N | Y | N | 4 |
| 2 TLE+HS | M | 27 | 3 | Y | N | Y | 4 |
| 3 TLE+HS | M | 26 | 16 | N | N | N | 1 |
| 4 TLE+HS | F | 39 | 18 | Y | N | Y | 30 |
| 5 TLE+HS | M | 20 | 1 | N | N | Y | 60 |
| 6 TLE+HS | F | 22 | 18 | Y | N | N | 12 |
| 7 TLE+HS | M | 18 | 2 | N | N | N | 3 |
| 8 TLE+HS | F | 32 | 2 | N | N | N | 2 |
| 9 TLE+HS | F | 26 | 6 | Y | N | Y | 1 |
| 10 TLE+HS | F | 29 | 13 | N | N | N | 1 |
| 11 TLE+HS | F | 31 | 12 | Y | N | N | 2 |
| 12 TLE+HS | M | 21 | 13 | N | N | N | 12 |
| 13 TLE+HS | F | 25 | 6 | Y | N | N | 10 |
| 14 TLE+HS | F | 27 | 15 | Y | N | N | 90 |
| 15 TLE+HS | F | 31 | 4 | Y | N | Y | 8 |
| 16 TLE+HS | M | 53 | 51 | N | N | Y | 90 |
| 17 TLE+HS | F | 34 | 25 | Y | N | Y | 60 |
| 18 TLE+HS | F | 36 | 7 | N | N | N | 20 |
| Continued |  |  |  |  |  |  |  |
| Num. | Duration (yr) | GTCS before surgery | AEDs before surgery | SEEG | HS | Resection tissue | FCD |
| 1 TLE-HS | 16 | N | VPA,OXC,LEV | N | N | R | 1 |
| 2 TLE-HS | 9 | N | OXC,LEV | N | N | L | 3 |
| 3 TLE-HS | 6 | Y | TPM,OXC | N | N | L | 3 |
| 4 TLE-HS | 18 | N | CBZ,VPA | N | N | L | 1 |
| 5 TLE-HS | 10 | N | PHT,PB,VPA | N | N | L | 1 |
| 6 TLE-HS | 5 | Y | OXC,GBP,LEV | N | N | R | 3 |
| 7 TLE-HS | 35 | Y | CBZ,VPA | N | N | L | 3 |
| 8 TLE-HS | 13 | Y | LEV,OXC,PB | N | N | L | 1 |
| 9 TLE-HS | 8 | N | CBZ,PHT,LTG | N | N | L | 1 |
| 10 TLE-HS | 19 | N | VPA,PB | N | N | R | 3 |
| 11 TLE-HS | 23 | Y | PB,PHT,VPA,CBZ, | N | N | L | 3 |
| 12 TLE-HS | 4 | N | OXC,GBP,LEV | N | N | R | 1 |
| 13 TLE-HS | 5 | Y | OXC,LTG,LEV | N | N | R | 1 |
| 14 TLE-HS | 2 | N | CBZ,GBP,PHT | N | N | L | 3 |
| 15 TLE-HS | 12 | Y | LEV,VPA,OXC | N | N | R | 3 |
| 16 TLE-HS | 15 | N | VPA,CLZ,PB,CBZ,TPM | N | N | R | 3 |
| 17 TLE-HS | 15 | N | PHT,VPA,CBZ,TPM | N | N | L | 2 |
| 18 TLE-HS | 9 | N | VPA,CBZ,LEV,CLZ,TPM | N | N | R | 3 |
| 1 TLE+HS | 11 | Y | VPA,CBZ | N | Y | R | 1 |
| 2 TLE+HS | 24 | Y | PHT,CBZ,VPA,TPM | N | Y | R | 3 |
| 3 TLE+HS | 10 | Y | TPM,OXC,LEV | N | Y | L | 1 |
| 4 TLE+HS | 21 | N | CBZ,LTG,CLZ | N | Y | L | 3 |
| 5 TLE+HS | 19 | N | PHT,CBZ | N | Y | L | 3 |
| 6 TLE+HS | 4 | Y | LTG,TPM,GBP | Y | Y | R | 1 |
| 7 TLE+HS | 16 | Y | VPA,PHT,PB | N | Y | L | 1 |
| 8 TLE+HS | 30 | Y | OXC,TPM | N | Y | R | 1 |
| 9 TLE+HS | 20 | Y | CBZ,TPM,PHT | N | Y | L | 3 |
| 10 TLE+HS | 16 | Y | PHT,TPM,CBZ | N | Y | L | 1 |
| 11 TLE+HS | 19 | Y | VPA,CBZ | N | Y | L | 1 |
| 12 TLE+HS | 8 | Y | CBZ,VPA | N | Y | R | 1 |
| 13 TLE+HS | 19 | N | CBZ,TPM,VPA | N | Y | L | 2 |
| 14 TLE+HS | 12 | Y | LTG,TPM,GBP | N | Y | L | 1 |
| 15 TLE+HS | 27 | Y | CBZ,VPA,PHT, | N | Y | L | 3 |
| 16 TLE+HS | 2 | N | TPM,LTG,LEV | N | Y | L | 3 |
| 17 TLE+HS | 9 | N | TPM,OXC,LEV | N | Y | L | 3 |
| 18 TLE+HS | 29 | Y | CBZ,OXC,VPA | N | Y | L | 1 |

F, female; M, male; Y, yes; N, no; L, left; R, right; IPI, initial precipitating incident (e.g., trauma, ischemic injury, encephalitis); CPS, complex partial seizure; GTCS, generalized tonic clonic seizure; SE, status epilepticus; HS, hippocampal sclerosis; AEDs, antiepileptic drugs; VPA, valproic acid; CBZ, carbamazepine; LTG, lamotrigine; OXC, oxcarbazepine; GBP, gabapentin; TPM, topamax; PHT, phenytoin; LEV, levetiracetam; PB, phenobarbital; SEEG, stereo-electroencephalography; FCD, focal cortical dysplasia.

**Table S4**. Differentially expressed 513 exosomal miRNAs in 6 mTLE+HS patients compared to 6 mTLE-HS controls by Illumina Hiseq2500 sequencing.

| Num. | miRNA | up/down | log_2_(patient/control) | p-value |
| --- | --- | --- | --- | --- |
| 1 | hsa-miR-184 | down | -4.9311 | *0.00064* |
| 2 | hsa-miR-219a-2-3p | up | 5.8573 | *0.00103* |
| 3 | hsa-miR-219b-5p | up | 5.9519 | *0.00228* |
| 4 | hsa-miR-3065-5p | up | 3.9192 | *0.00531* |
| 5 | hsa-miR-4667-5p | down | -4.4686 | *0.00658* |
| 6 | hsa-miR-3176 | up | 1.2277 | *0.0093* |
| 7 | hsa-miR-493-5p | up | 1.2723 | *0.01* |
| 8 | hsa-miR-214-3p | up | 1.4368 | *0.0146* |
| 9 | hsa-miR-548ae-5p | down | -4.4692 | *0.01479* |
| 10 | hsa-miR-548ad-5p | down | -4.4692 | *0.01486* |
| 11 | hsa-miR-6866-5p | down | -4.2012 | *0.01511* |
| 12 | hsa-miR-383-5p | up | 4.2987 | *0.0157* |
| 13 | hsa-miR-4746-5p | up | 1.0194 | *0.0157* |
| 14 | hsa-miR-889-3p | up | 3.9307 | *0.0164* |
| 15 | hsa-miR-450a-5p | down | -2.5281 | *0.01982* |
| 16 | hsa-miR-4449 | up | 4.4976 | *0.0201* |
| 17 | hsa-miR-676-3p | up | 4.2979 | *0.02086* |
| 18 | hsa-miR-335-3p | down | -4.3861 | *0.0214* |
| 19 | hsa-miR-4741 | up | 2.4838 | *0.02348* |
| 20 | hsa-miR-421 | down | -2.9797 | *0.0238* |
| 21 | hsa-miR-4658 | up | 4.7834 | *0.02424* |
| 22 | hsa-miR-4734 | up | 2.5491 | *0.02472* |
| 23 | hsa-miR-6821-5p | up | 5.0021 | *0.02546* |
| 24 | hsa-miR-3131 | up | 4.4114 | *0.02597* |
| 25 | hsa-miR-4750-5p | down | -2.0916 | *0.02676* |
| 26 | hsa-miR-6852-3p | down | -4.0618 | *0.02843* |
| 27 | hsa-miR-129-5p | up | 1.2379 | *0.02858* |
| 28 | hsa-miR-4717-3p | up | 4.0652 | *0.03002* |
| 29 | hsa-miR-181a-2-3p | down | -1.6923 | *0.03033* |
| 30 | hsa-miR-301a-5p | down | -4.7043 | *0.03362* |
| 31 | hsa-miR-6740-5p | up | 3.7004 | *0.03384* |
| 32 | hsa-miR-4676-3p | down | -4.1875 | *0.03522* |
| 33 | hsa-miR-4516 | down | -1.7946 | *0.03657* |
| 34 | hsa-miR-3200-5p | up | 1.369 | *0.03985* |
| 35 | hsa-miR-2276-3p | up | 2.2197 | *0.0416* |
| 36 | hsa-miR-219b-3p | up | 3.5969 | *0.04162* |
| 37 | hsa-miR-219a-5p | up | 3.5969 | *0.04171* |
| 38 | hsa-miR-4638-5p | down | -2.9727 | *0.04193* |
| 39 | hsa-miR-6837-3p | down | -3.8589 | *0.04313* |
| 40 | hsa-miR-451a | down | -2.0252 | *0.04439* |
| 41 | hsa-miR-154-3p | up | 4.2972 | *0.04677* |
| 42 | hsa-miR-34c-5p | up | 2.0179 | *0.04952* |
| 43 | hsa-miR-5695 | down | -3.9533 | *0.0504* |
| 44 | hsa-miR-4800-5p | down | -2.9602 | *0.05093* |
| 45 | hsa-miR-30b-3p | down | -1.8441 | *0.05143* |
| 46 | hsa-miR-548az-5p | down | -1.9827 | *0.05189* |
| 47 | hsa-miR-194-3p | down | -3.1949 | *0.05477* |
| 48 | hsa-miR-4773 | up | 2.6661 | *0.05509* |
| 49 | hsa-miR-1-3p | down | -1.7236 | *0.05591* |
| 50 | hsa-miR-548an | down | -3.9011 | *0.05813* |
| 51 | hsa-miR-4539 | up | 3.7719 | *0.0584* |
| 52 | hsa-miR-3168 | down | -3.6264 | *0.05889* |
| 53 | hsa-miR-3960 | down | -1.5043 | *0.06047* |
| 54 | hsa-miR-1228-5p | up | 1.8069 | *0.06083* |
| 55 | hsa-miR-4634 | up | 2.612 | *0.0679* |
| 56 | hsa-miR-579-5p | up | 1.2993 | *0.06791* |
| 57 | hsa-miR-6889-5p | down | -3.8104 | *0.06931* |
| 58 | hsa-miR-580-3p | down | -3.7975 | *0.06994* |
| 59 | hsa-miR-885-3p | up | 1.6802 | *0.071* |
| 60 | hsa-miR-296-3p | up | 1.2643 | *0.07172* |
| 61 | hsa-miR-3135b | down | -1.8107 | *0.07209* |
| 62 | hsa-miR-4784 | up | 2.6683 | *0.07368* |
| 63 | hsa-miR-4492 | up | 1.3659 | *0.07398* |
| 64 | hsa-miR-5189-5p | down | -3.8327 | *0.07427* |
| 65 | hsa-miR-641 | down | -3.5581 | *0.07693* |
| 66 | hsa-miR-218-5p | up | 1.3399 | *0.08143* |
| 67 | hsa-miR-3611 | down | -2.6965 | *0.08598* |
| 68 | hsa-miR-7114-5p | up | 1.9888 | *0.08713* |
| 69 | hsa-miR-1275 | down | -2.5534 | *0.09113* |
| 70 | hsa-miR-6843-3p | down | -2.6149 | *0.09233* |
| 71 | hsa-miR-1343-3p | down | -3.8738 | *0.09267* |
| 72 | hsa-miR-219a-1-3p | down | -2.5063 | *0.09284* |
| 73 | hsa-miR-429 | down | -3.7981 | *0.09432* |
| 74 | hsa-miR-126-3p | down | -1.9743 | *0.09523* |
| 75 | hsa-miR-4687-3p | down | -3.5981 | *0.09609* |
| 76 | hsa-miR-6745 | up | 3.5536 | *0.09641* |
| 77 | hsa-miR-154-5p | up | 3.1763 | *0.09652* |
| 78 | hsa-miR-4523 | down | -3.2063 | *0.10061* |
| 79 | hsa-miR-4646-5p | up | 1.2524 | *0.10227* |
| 80 | hsa-miR-146a-3p | down | -3.8629 | *0.10241* |
| 81 | hsa-miR-26b-5p | down | -1.6771 | *0.10586* |
| 82 | hsa-miR-4726-5p | up | 2.0306 | *0.11251* |
| 83 | hsa-miR-29c-3p | down | -2.2086 | *0.11546* |
| 84 | hsa-miR-671-5p | up | 1.1193 | *0.11928* |
| 85 | hsa-miR-7108-5p | down | -3.9809 | *0.12412* |
| 86 | hsa-miR-487b-3p | up | 1.2363 | *0.12661* |
| 87 | hsa-miR-6855-5p | down | -3.2771 | *0.13058* |
| 88 | hsa-miR-3653-5p | up | 2.9317 | *0.13157* |
| 89 | hsa-miR-4489 | down | -2.0138 | *0.13165* |
| 90 | hsa-miR-7847-3p | down | -1.9325 | *0.13537* |
| 91 | hsa-miR-7855-5p | up | 2.9653 | *0.1354* |
| 92 | hsa-miR-6727-5p | down | -3.546 | *0.13857* |
| 93 | hsa-miR-3690 | down | -3.4529 | *0.14038* |
| 94 | hsa-miR-501-5p | up | 1.5516 | *0.14586* |
| 95 | hsa-miR-4487 | down | -2.132 | *0.14606* |
| 96 | hsa-miR-202-3p | down | -2.9628 | *0.14643* |
| 97 | hsa-miR-3127-3p | down | -3.237 | *0.15066* |
| 98 | hsa-miR-148b-5p | down | -1.9951 | *0.15113* |
| 99 | hsa-miR-6813-3p | down | -2.9635 | *0.15403* |
| 100 | hsa-miR-1273e | up | 1.1836 | *0.15695* |
| 101 | hsa-miR-1299 | down | -1.4177 | *0.15849* |
| 102 | hsa-miR-3184-3p | down | -1.0293 | *0.16193* |
| 103 | hsa-miR-423-5p | down | -1.0292 | *0.16213* |
| 104 | hsa-miR-3653-3p | down | -1.1731 | *0.16389* |
| 105 | hsa-miR-6754-5p | down | -3.2348 | *0.16671* |
| 106 | hsa-miR-4676-5p | down | -2.5391 | *0.16926* |
| 107 | hsa-miR-24-2-5p | down | -1.4078 | *0.16989* |
| 108 | hsa-miR-3195 | up | 1.162 | *0.17079* |
| 109 | hsa-miR-6852-5p | down | -1.2417 | *0.17088* |
| 110 | hsa-miR-1273f | down | -1.1705 | *0.17464* |
| 111 | hsa-miR-32-5p | down | -3.3025 | *0.17706* |
| 112 | hsa-miR-485-3p | up | 2.9561 | *0.1772* |
| 113 | hsa-miR-3916 | up | 2.1257 | *0.17766* |
| 114 | hsa-miR-6772-5p | down | -3.6599 | *0.17814* |
| 115 | hsa-miR-95-3p | up | 1.8143 | *0.17881* |
| 116 | hsa-miR-3188 | up | 2.3884 | *0.18051* |
| 117 | hsa-miR-3173-5p | up | 2.0177 | *0.18407* |
| 118 | hsa-miR-550b-2-5p | down | -1.9792 | *0.18434* |
| 119 | hsa-miR-550a-3p | down | -1.9792 | *0.18464* |
| 120 | hsa-miR-26a-5p | down | -1.683 | *0.18565* |
| 121 | hsa-miR-3200-3p | up | 1.7946 | *0.18893* |
| 122 | hsa-miR-6874-3p | down | -2.9855 | *0.18932* |
| 123 | hsa-miR-338-3p | up | 2.6206 | *0.19494* |
| 124 | hsa-miR-7846-3p | down | -2.5826 | *0.19782* |
| 125 | hsa-miR-5680 | down | -3.1194 | *0.20028* |
| 126 | hsa-miR-29b-2-5p | up | 1.2206 | *0.20118* |
| 127 | hsa-miR-345-3p | up | 1.1032 | *0.2026* |
| 128 | hsa-miR-1343-5p | up | 2.9184 | *0.20803* |
| 129 | hsa-miR-5703 | down | -2.6415 | *0.20922* |
| 130 | hsa-miR-4649-5p | down | -2.6415 | *0.20958* |
| 131 | hsa-miR-28-5p | down | -1.6742 | *0.20982* |
| 132 | hsa-miR-3154 | down | -2.6415 | *0.21033* |
| 133 | hsa-miR-4647 | down | -1.7879 | *0.21072* |
| 134 | hsa-miR-3157-3p | up | 1.7432 | *0.21637* |
| 135 | hsa-miR-3150a-3p | down | -1.4226 | *0.21711* |
| 136 | hsa-miR-30c-5p | down | -1.4851 | *0.223* |
| 137 | hsa-miR-4669 | down | -1.8672 | *0.22487* |
| 138 | hsa-miR-155-5p | down | -1.2634 | *0.22615* |
| 139 | hsa-miR-6862-5p | down | -1.6165 | *0.2284* |
| 140 | hsa-miR-4762-3p | down | -3.1144 | *0.23269* |
| 141 | hsa-miR-509-3-5p | up | 2.6017 | *0.23557* |
| 142 | hsa-miR-6857-3p | up | 2.6276 | *0.23624* |
| 143 | hsa-miR-4677-3p | down | -2.1459 | *0.23845* |
| 144 | hsa-miR-1273c | down | -1.2325 | *0.23902* |
| 145 | hsa-miR-526b-5p | down | -3.3001 | *0.2394* |
| 146 | hsa-miR-659-5p | down | -1.2384 | *0.24073* |
| 147 | hsa-miR-200b-3p | down | -2.2433 | *0.24324* |
| 148 | hsa-miR-6833-3p | up | 1.6496 | *0.24507* |
| 149 | hsa-miR-6861-5p | down | -3.0318 | *0.24526* |
| 150 | hsa-miR-6819-5p | up | 2.6984 | *0.24538* |
| 151 | hsa-miR-940 | down | -3.0662 | *0.24624* |
| 152 | hsa-miR-30b-5p | down | -1.5377 | *0.24752* |
| 153 | hsa-miR-4507 | up | 2.1827 | *0.24794* |
| 154 | hsa-miR-6829-5p | down | -3.3118 | *0.24847* |
| 155 | hsa-miR-1909-5p | down | -3.1457 | *0.24891* |
| 156 | hsa-miR-3622a-5p | up | 2.1667 | *0.24981* |
| 157 | hsa-miR-483-5p | up | 1.0525 | *0.25025* |
| 158 | hsa-miR-627-5p | down | -1.69 | *0.25053* |
| 159 | hsa-miR-4535 | down | -1.4148 | *0.25101* |
| 160 | hsa-miR-212-5p | down | -2.3737 | *0.25544* |
| 161 | hsa-miR-181c-3p | down | -1.0237 | *0.25615* |
| 162 | hsa-miR-7975 | down | -3.4867 | *0.25737* |
| 163 | hsa-miR-6724-5p | down | -2.4141 | *0.25738* |
| 164 | hsa-miR-378j | up | 1.7109 | *0.25781* |
| 165 | hsa-miR-6511a-3p | down | -2.6276 | *0.25793* |
| 166 | hsa-miR-4484 | up | 2.5969 | *0.25869* |
| 167 | hsa-miR-216a-5p | up | 2.5969 | *0.25922* |
| 168 | hsa-miR-942-5p | down | -1.1734 | *0.25993* |
| 169 | hsa-miR-6516-3p | down | -3.4612 | *0.26328* |
| 170 | hsa-miR-454-5p | down | -3.5274 | *0.27159* |
| 171 | hsa-miR-6793-3p | down | -2.9579 | *0.272* |
| 172 | hsa-miR-4488 | down | -1.2818 | *0.27223* |
| 173 | hsa-miR-6853-3p | up | 2.2172 | *0.27418* |
| 174 | hsa-miR-203a-3p | down | -1.8238 | *0.27638* |
| 175 | hsa-miR-4430 | down | -1.0184 | *0.27785* |
| 176 | hsa-miR-6502-5p | up | 1.4615 | *0.2787* |
| 177 | hsa-miR-450b-5p | down | -1.4846 | *0.27895* |
| 178 | hsa-miR-6859-5p | down | -1.5658 | *0.28009* |
| 179 | hsa-miR-1281 | down | -2.7994 | *0.28519* |
| 180 | hsa-miR-574-5p | up | 1.4003 | *0.28712* |
| 181 | hsa-miR-500b-3p | down | -1.6574 | *0.28941* |
| 182 | hsa-miR-132-5p | down | -1.5906 | *0.28976* |
| 183 | hsa-let-7d-5p | down | -1.6399 | *0.29311* |
| 184 | hsa-miR-1304-5p | down | -1.4119 | *0.29513* |
| 185 | hsa-miR-221-5p | down | -1.4008 | *0.29793* |
| 186 | hsa-miR-873-5p | down | -2.5705 | *0.30098* |
| 187 | hsa-miR-6787-5p | down | -2.5509 | *0.30298* |
| 188 | hsa-miR-2115-5p | down | -1.3477 | *0.30324* |
| 189 | hsa-miR-3133 | up | 1.6941 | *0.30341* |
| 190 | hsa-miR-3177-3p | down | -1.1569 | *0.30357* |
| 191 | hsa-miR-1284 | down | -3.3447 | *0.30418* |
| 192 | hsa-miR-192-5p | down | -1.4746 | *0.31484* |
| 193 | hsa-let-7f-2-3p | down | -2.5311 | *0.31644* |
| 194 | hsa-miR-199a-5p | down | -1.3562 | *0.31676* |
| 195 | hsa-miR-6763-5p | up | 1.1894 | *0.32067* |
| 196 | hsa-miR-190a-5p | down | -1.2171 | *0.3212* |
| 197 | hsa-miR-222-5p | down | -1.5592 | *0.32816* |
| 198 | hsa-miR-6728-3p | up | 2.1827 | *0.33155* |
| 199 | hsa-miR-2355-5p | up | 2.2327 | *0.33329* |
| 200 | hsa-miR-4707-3p | up | 1.193 | *0.33335* |
| 201 | hsa-miR-483-3p | up | 1.0469 | *0.33351* |
| 202 | hsa-miR-664b-5p | down | -1.7508 | *0.33363* |
| 203 | hsa-miR-5006-3p | down | -3.4816 | *0.33483* |
| 204 | hsa-miR-6741-3p | down | -2.4642 | *0.33505* |
| 205 | hsa-miR-6511b-5p | down | -1.8593 | *0.33699* |
| 206 | hsa-miR-4746-3p | up | 2.1795 | *0.33855* |
| 207 | hsa-miR-598-5p | down | -2.7277 | *0.33898* |
| 208 | hsa-miR-142-3p | down | -1.3709 | *0.33926* |
| 209 | hsa-miR-222-3p | down | -1.1688 | *0.34019* |
| 210 | hsa-miR-195-5p | down | -2.852 | *0.34157* |
| 211 | hsa-miR-6809-5p | down | -2.6369 | *0.34277* |
| 212 | hsa-miR-6716-3p | down | -2.6369 | *0.34304* |
| 213 | hsa-miR-4731-5p | down | -2.6369 | *0.34352* |
| 214 | hsa-miR-6878-5p | down | -2.8197 | *0.34488* |
| 215 | hsa-miR-523-5p | down | -2.1699 | *0.34544* |
| 216 | hsa-miR-519c-5p | down | -2.1699 | *0.34546* |
| 217 | hsa-miR-518e-5p | down | -2.1699 | *0.34557* |
| 218 | hsa-miR-519b-5p | down | -2.1699 | *0.34565* |
| 219 | hsa-miR-4742-5p | down | -2.8278 | *0.34565* |
| 220 | hsa-miR-522-5p | down | -2.1699 | *0.34599* |
| 221 | hsa-miR-519a-5p | down | -2.1699 | *0.34607* |
| 222 | hsa-miR-4710 | down | -2.8359 | *0.34725* |
| 223 | hsa-miR-548q | down | -3.1268 | *0.34808* |
| 224 | hsa-miR-615-3p | up | 1.5708 | *0.3485* |
| 225 | hsa-miR-1258 | down | -2.2079 | *0.3509* |
| 226 | hsa-miR-4500 | down | -2.8679 | *0.35213* |
| 227 | hsa-miR-6770-3p | down | -1.5956 | *0.35317* |
| 228 | hsa-miR-4306 | up | 2.1699 | *0.35359* |
| 229 | hsa-miR-642a-5p | up | 2.1699 | *0.35398* |
| 230 | hsa-miR-3127-5p | down | -1.846 | *0.35819* |
| 231 | hsa-miR-6833-5p | up | 2.1985 | *0.35896* |
| 232 | hsa-miR-4648 | down | -3.0875 | *0.36152* |
| 233 | hsa-miR-548al | down | -3.106 | *0.36231* |
| 234 | hsa-miR-3161 | down | -2.6253 | *0.36241* |
| 235 | hsa-miR-769-3p | up | 1.4098 | *0.36995* |
| 236 | hsa-miR-23c | down | -2.5705 | *0.37298* |
| 237 | hsa-miR-1468-5p | down | -2.2766 | *0.37685* |
| 238 | hsa-miR-516b-5p | down | -1.8386 | *0.38433* |
| 239 | hsa-miR-4732-3p | down | -1.018 | *0.38445* |
| 240 | hsa-miR-140-5p | down | -1.5972 | *0.38683* |
| 241 | hsa-miR-6808-3p | down | -2.5993 | *0.39017* |
| 242 | hsa-miR-487b-5p | down | -2.5993 | *0.39027* |
| 243 | hsa-miR-4743-5p | down | -1.5587 | *0.39235* |
| 244 | hsa-miR-3199 | up | 1.1932 | *0.39342* |
| 245 | hsa-miR-21-5p | down | -1.237 | *0.3966* |
| 246 | hsa-miR-223-3p | down | -1.2537 | *0.39948* |
| 247 | hsa-miR-99a-5p | down | -1.2521 | *0.40044* |
| 248 | hsa-miR-130b-5p | down | -1.3214 | *0.40129* |
| 249 | hsa-miR-6515-3p | up | 2.0354 | *0.40401* |
| 250 | hsa-miR-628-5p | down | -2.814 | *0.41168* |
| 251 | hsa-miR-214-5p | down | -1.4618 | *0.4132* |
| 252 | hsa-miR-335-5p | down | -1.4408 | *0.41415* |
| 253 | hsa-miR-548ay-5p | down | -1.7175 | *0.41615* |
| 254 | hsa-miR-411-5p | down | -1.0472 | *0.41629* |
| 255 | hsa-miR-6816-3p | up | 2.2388 | *0.41769* |
| 256 | hsa-miR-6738-3p | up | 2.2388 | *0.41776* |
| 257 | hsa-miR-371b-3p | up | 2.2388 | *0.41802* |
| 258 | hsa-miR-660-3p | up | 2.2388 | *0.41841* |
| 259 | hsa-miR-6130 | down | -1.3452 | *0.41886* |
| 260 | hsa-miR-616-3p | up | 1.3945 | *0.41942* |
| 261 | hsa-miR-6746-5p | up | 2.2327 | *0.41993* |
| 262 | hsa-miR-18b-3p | up | 2.2327 | *0.42023* |
| 263 | hsa-miR-3654 | up | 2.2327 | *0.42027* |
| 264 | hsa-miR-6516-5p | down | -1.9749 | *0.4215* |
| 265 | hsa-miR-200c-3p | down | -1.6238 | *0.42335* |
| 266 | hsa-miR-5195-3p | up | 2.6531 | *0.42661* |
| 267 | hsa-miR-133b | down | -2.3964 | *0.42861* |
| 268 | hsa-miR-3146 | down | -3.007 | *0.43376* |
| 269 | hsa-miR-4743-3p | up | 2.1375 | *0.43646* |
| 270 | hsa-miR-365a-5p | down | -1.4663 | *0.43677* |
| 271 | hsa-miR-4303 | up | 1.5945 | *0.44065* |
| 272 | hsa-miR-4513 | up | 1.5945 | *0.44076* |
| 273 | hsa-miR-4294 | up | 1.5945 | *0.44106* |
| 274 | hsa-miR-607 | up | 1.5945 | *0.44151* |
| 275 | hsa-miR-1301-3p | down | -1.1726 | *0.44284* |
| 276 | hsa-miR-3163 | down | -2.5993 | *0.4466* |
| 277 | hsa-miR-6840-3p | down | -2.2203 | *0.44763* |
| 278 | hsa-miR-4749-5p | up | 1.6323 | *0.4489* |
| 279 | hsa-miR-4717-5p | up | 1.6323 | *0.44917* |
| 280 | hsa-miR-4514 | up | 1.6323 | *0.44946* |
| 281 | hsa-miR-4491 | up | 1.6323 | *0.4499* |
| 282 | hsa-miR-504-3p | up | 1.6323 | *0.45028* |
| 283 | hsa-miR-152-5p | up | 1.6323 | *0.45049* |
| 284 | hsa-miR-374c-3p | down | -1.3687 | *0.45244* |
| 285 | hsa-miR-374b-5p | down | -1.3687 | *0.45264* |
| 286 | hsa-miR-4482-5p | up | 1.5378 | *0.45481* |
| 287 | hsa-miR-378a-5p | down | -1.33 | *0.45964* |
| 288 | hsa-miR-224-5p | down | -1.4145 | *0.45965* |
| 289 | hsa-miR-935 | down | -2.2898 | *0.45977* |
| 290 | hsa-miR-6875-5p | down | -3.0891 | *0.46148* |
| 291 | hsa-miR-146b-5p | down | -1.0006 | *0.4617* |
| 292 | hsa-miR-450a-2-3p | up | 1.5303 | *0.46882* |
| 293 | hsa-miR-766-5p | down | -1.0961 | *0.4703* |
| 294 | hsa-miR-6882-5p | down | -2.3568 | *0.4716* |
| 295 | hsa-miR-767-5p | up | 1.9211 | *0.47371* |
| 296 | hsa-miR-4425 | up | 2.0809 | *0.47486* |
| 297 | hsa-miR-1270 | down | -1.5609 | *0.47513* |
| 298 | hsa-miR-4533 | down | -1.0148 | *0.47557* |
| 299 | hsa-miR-4419b | up | 1.1167 | *0.47622* |
| 300 | hsa-miR-4521 | down | -2.3219 | *0.47863* |
| 301 | hsa-miR-520a-3p | down | -2.3072 | *0.48034* |
| 302 | hsa-miR-16-5p | down | -1.1464 | *0.48374* |
| 303 | hsa-miR-181a-3p | down | -1.0549 | *0.48573* |
| 304 | hsa-miR-1285-5p | down | -1.1494 | *0.5032* |
| 305 | hsa-miR-5001-3p | up | 1.4138 | *0.50354* |
| 306 | hsa-miR-550a-3-5p | down | -1.1226 | *0.51313* |
| 307 | hsa-miR-541-3p | down | -2.8602 | *0.51517* |
| 308 | hsa-let-7c-5p | down | -1.1033 | *0.51775* |
| 309 | hsa-miR-625-3p | down | -1.2041 | *0.52383* |
| 310 | hsa-miR-205-5p | down | -1.0628 | *0.52468* |
| 311 | hsa-miR-548j-5p | down | -1.4265 | *0.52602* |
| 312 | hsa-miR-27a-3p | down | -1.0775 | *0.52604* |
| 313 | hsa-miR-217 | down | -2.0023 | *0.52673* |
| 314 | hsa-miR-5189-3p | down | -1.9768 | *0.52771* |
| 315 | hsa-miR-3192-5p | down | -2.7661 | *0.52828* |
| 316 | hsa-miR-455-3p | down | -2.7172 | *0.53121* |
| 317 | hsa-miR-4642 | down | -2.1077 | *0.53455* |
| 318 | hsa-miR-5003-3p | down | -2.1077 | *0.53475* |
| 319 | hsa-miR-4709-3p | down | -2.0114 | *0.53615* |
| 320 | hsa-miR-887-3p | down | -1.1281 | *0.53623* |
| 321 | hsa-miR-4745-5p | down | -2.3046 | *0.5371* |
| 322 | hsa-miR-7152-3p | down | -1.5351 | *0.53942* |
| 323 | hsa-miR-5004-5p | up | 1.6041 | *0.54016* |
| 324 | hsa-miR-6509-3p | down | -1.7953 | *0.5405* |
| 325 | hsa-miR-3121-3p | down | -1.5993 | *0.54333* |
| 326 | hsa-miR-3201 | up | 1.6229 | *0.54388* |
| 327 | hsa-miR-4317 | down | -1.6508 | *0.54391* |
| 328 | hsa-miR-6858-5p | down | -1.6289 | *0.55002* |
| 329 | hsa-miR-2115-3p | down | -1.2708 | *0.55076* |
| 330 | hsa-miR-5090 | down | -2.511 | *0.55129* |
| 331 | hsa-miR-7974 | up | 1.5753 | *0.552* |
| 332 | hsa-miR-3685 | down | -2.541 | *0.555* |
| 333 | hsa-miR-3678-3p | down | -2.541 | *0.55502* |
| 334 | hsa-miR-188-5p | down | -1.4653 | *0.55507* |
| 335 | hsa-miR-3622b-3p | up | 1.5753 | *0.55668* |
| 336 | hsa-miR-6755-5p | down | -2.1473 | *0.5592* |
| 337 | hsa-miR-194-5p | down | -1.0882 | *0.5613* |
| 338 | hsa-miR-9-3p | down | -1.9116 | *0.56675* |
| 339 | hsa-miR-6767-5p | down | -1.9001 | *0.56868* |
| 340 | hsa-miR-6817-3p | up | 1.1653 | *0.5719* |
| 341 | hsa-miR-4772-5p | up | 1.1607 | *0.5755* |
| 342 | hsa-miR-6775-3p | up | 1.1607 | *0.5758* |
| 343 | hsa-miR-6503-3p | down | -1.1848 | *0.57987* |
| 344 | hsa-miR-943 | up | 2.2697 | *0.58437* |
| 345 | hsa-miR-548f-5p | down | -1.4067 | *0.58624* |
| 346 | hsa-miR-548av-3p | down | -1.9862 | *0.59143* |
| 347 | hsa-miR-4463 | up | 2.2768 | *0.59537* |
| 348 | hsa-miR-24-3p | down | -1.0906 | *0.59551* |
| 349 | hsa-miR-552-3p | down | -2.6285 | *0.59737* |
| 350 | hsa-miR-5581-3p | up | 1.6508 | *0.59932* |
| 351 | hsa-miR-3180-5p | up | 1.6508 | *0.59937* |
| 352 | hsa-miR-548l | up | 1.6508 | *0.59967* |
| 353 | hsa-miR-3120-3p | down | -1.3081 | *0.59984* |
| 354 | hsa-miR-1226-3p | up | 1.6508 | *0.59986* |
| 355 | hsa-miR-34b-3p | up | 1.6508 | *0.59993* |
| 356 | hsa-miR-7110-5p | up | 1.6462 | *0.60098* |
| 357 | hsa-miR-340-3p | down | -1.4971 | *0.60119* |
| 358 | hsa-miR-6735-3p | up | 1.6462 | *0.60122* |
| 359 | hsa-miR-4677-5p | up | 1.6462 | *0.60166* |
| 360 | hsa-miR-1179 | up | 1.6462 | *0.60184* |
| 361 | hsa-miR-548k | down | -1.2762 | *0.60946* |
| 362 | hsa-miR-6748-5p | down | -1.4884 | *0.6113* |
| 363 | hsa-miR-3944-3p | down | -1.5211 | *0.61606* |
| 364 | hsa-miR-4712-3p | down | -1.5211 | *0.61613* |
| 365 | hsa-miR-6819-3p | down | -1.5211 | *0.61625* |
| 366 | hsa-miR-6739-5p | down | -1.5211 | *0.61637* |
| 367 | hsa-miR-3667-3p | down | -1.5211 | *0.61658* |
| 368 | hsa-miR-6771-5p | down | -1.5211 | *0.61663* |
| 369 | hsa-miR-3162-5p | down | -1.5211 | *0.61701* |
| 370 | hsa-miR-548au-5p | down | -2.4803 | *0.61813* |
| 371 | hsa-miR-5587-5p | down | -2.4803 | *0.61837* |
| 372 | hsa-miR-6818-3p | down | -2.4803 | *0.6185* |
| 373 | hsa-miR-4747-5p | down | -2.4803 | *0.61855* |
| 374 | hsa-miR-6840-5p | up | 1.5509 | *0.61879* |
| 375 | hsa-miR-6846-5p | up | 1.5509 | *0.61883* |
| 376 | hsa-miR-3651 | up | 1.5509 | *0.61884* |
| 377 | hsa-miR-3944-5p | down | -2.4803 | *0.61885* |
| 378 | hsa-miR-4722-5p | up | 1.5509 | *0.61891* |
| 379 | hsa-miR-3165 | down | -2.4803 | *0.61909* |
| 380 | hsa-miR-499b-3p | down | -2.3521 | *0.62017* |
| 381 | hsa-miR-6820-5p | down | -1.286 | *0.62203* |
| 382 | hsa-miR-182-3p | up | 1.5945 | *0.6235* |
| 383 | hsa-miR-6766-5p | down | -2.8032 | *0.62553* |
| 384 | hsa-miR-145-3p | down | -1.2546 | *0.62553* |
| 385 | hsa-miR-548am-5p | down | -2.8032 | *0.6257* |
| 386 | hsa-miR-6789-3p | down | -1.6369 | *0.6258* |
| 387 | hsa-miR-548o-5p | down | -2.8032 | *0.62603* |
| 388 | hsa-miR-3183 | down | -1.6369 | *0.62616* |
| 389 | hsa-miR-299-3p | down | -1.6369 | *0.62646* |
| 390 | hsa-miR-3166 | down | -1.6369 | *0.62646* |
| 391 | hsa-miR-3144-5p | down | -2.8032 | *0.62647* |
| 392 | hsa-miR-3138 | down | -2.8032 | *0.62667* |
| 393 | hsa-miR-1909-3p | down | -2.8032 | *0.62709* |
| 394 | hsa-miR-627-3p | down | -2.8032 | *0.62718* |
| 395 | hsa-miR-548c-5p | down | -2.8032 | *0.62738* |
| 396 | hsa-miR-556-5p | down | -2.8032 | *0.62739* |
| 397 | hsa-miR-4772-3p | down | -1.5259 | *0.62877* |
| 398 | hsa-miR-7152-5p | down | -2.1077 | *0.62941* |
| 399 | hsa-miR-4674 | down | -2.1077 | *0.62964* |
| 400 | hsa-miR-4505 | down | -2.1077 | *0.6298* |
| 401 | hsa-miR-581 | down | -2.1827 | *0.6299* |
| 402 | hsa-miR-1228-3p | down | -2.1077 | *0.63005* |
| 403 | hsa-miR-4762-5p | down | -2.1827 | *0.63011* |
| 404 | hsa-miR-590-3p | down | -2.1077 | *0.63046* |
| 405 | hsa-miR-7843-3p | down | -1.7049 | *0.63576* |
| 406 | hsa-miR-4721 | down | -1.7049 | *0.63579* |
| 407 | hsa-miR-6818-5p | down | -1.7049 | *0.63582* |
| 408 | hsa-miR-6749-3p | down | -1.7049 | *0.63584* |
| 409 | hsa-miR-4482-3p | down | -1.7049 | *0.63592* |
| 410 | hsa-miR-4672 | down | -1.7049 | *0.63597* |
| 411 | hsa-miR-3925-3p | down | -1.7049 | *0.63649* |
| 412 | hsa-miR-183-3p | down | -1.7049 | *0.63662* |
| 413 | hsa-let-7f-5p | down | -1.0637 | *0.64097* |
| 414 | hsa-miR-6764-5p | down | -1.7049 | *0.64473* |
| 415 | hsa-miR-3120-5p | down | -1.7798 | *0.64836* |
| 416 | hsa-let-7a-5p | down | -1.1482 | *0.65298* |
| 417 | hsa-miR-6747-3p | down | -1.3538 | *0.65776* |
| 418 | hsa-miR-181c-5p | down | -1.1008 | *0.65897* |
| 419 | hsa-miR-4661-5p | down | -1.0951 | *0.66027* |
| 420 | hsa-miR-3605-5p | down | -1.5478 | *0.6631* |
| 421 | hsa-miR-191-3p | down | -1.135 | *0.66337* |
| 422 | hsa-miR-122-5p | down | -1.0163 | *0.66477* |
| 423 | hsa-miR-363-3p | down | -1.0782 | *0.66484* |
| 424 | hsa-miR-29a-5p | down | -1.3427 | *0.66661* |
| 425 | hsa-miR-7854-3p | down | -1.3718 | *0.67789* |
| 426 | hsa-miR-885-5p | down | -1.3754 | *0.67872* |
| 427 | hsa-miR-128-3p | down | -1.0191 | *0.68173* |
| 428 | hsa-miR-33a-5p | down | -2.2793 | *0.68502* |
| 429 | hsa-miR-410-3p | up | 1.6071 | *0.69597* |
| 430 | hsa-miR-542-5p | down | -2.0695 | *0.6981* |
| 431 | hsa-miR-5584-5p | down | -2.1771 | *0.69974* |
| 432 | hsa-miR-3613-3p | up | 1.5278 | *0.70065* |
| 433 | hsa-miR-3918 | down | -1.5571 | *0.70301* |
| 434 | hsa-miR-4645-3p | up | 1.5754 | *0.70433* |
| 435 | hsa-miR-548a-3p | down | -2.3121 | *0.70785* |
| 436 | hsa-miR-323a-5p | up | 1.5247 | *0.70909* |
| 437 | hsa-miR-4781-3p | up | 1.4651 | *0.71042* |
| 438 | hsa-let-7e-5p | down | -1.0807 | *0.71882* |
| 439 | hsa-miR-409-5p | down | -1.2794 | *0.73178* |
| 440 | hsa-miR-548u | down | -1.4884 | *0.74683* |
| 441 | hsa-miR-675-5p | down | -1.9486 | *0.74993* |
| 442 | hsa-miR-548t-5p | up | 1.6215 | *0.75001* |
| 443 | hsa-miR-1295b-3p | down | -1.9486 | *0.75009* |
| 444 | hsa-let-7f-1-3p | down | -1.9486 | *0.7503* |
| 445 | hsa-miR-3181 | up | 1.0785 | *0.75033* |
| 446 | hsa-miR-377-3p | up | 1.0468 | *0.75332* |
| 447 | hsa-miR-6802-5p | up | 1.0377 | *0.75426* |
| 448 | hsa-miR-34a-5p | down | -1.6391 | *0.75548* |
| 449 | hsa-miR-589-3p | down | -1.6391 | *0.75575* |
| 450 | hsa-miR-4660 | down | -1.2561 | *0.76412* |
| 451 | hsa-miR-1304-3p | down | -1.0569 | *0.77974* |
| 452 | hsa-miR-98-5p | down | -1.1053 | *0.78779* |
| 453 | hsa-miR-4485-5p | down | -1.9765 | *0.79438* |
| 454 | hsa-miR-4755-5p | down | -1.5335 | *0.82379* |
| 455 | hsa-miR-2861 | up | 1.5411 | *0.82795* |
| 456 | hsa-miR-6772-3p | down | -1.1529 | *0.83813* |
| 457 | hsa-miR-203b-5p | down | -1.0333 | *0.88336* |
| 458 | hsa-miR-29b-3p | down | -1.3577 | *0.91546* |
| 459 | hsa-miR-1229-3p | down | -1.4464 | *1* |
| 460 | hsa-miR-1295a | up | 1.0562 | *1* |
| 461 | hsa-miR-3150b-5p | down | -1.4835 | *1* |
| 462 | hsa-miR-4473 | down | -1.5131 | *1* |
| 463 | hsa-miR-548x-5p | down | -1.4965 | *1* |
| 464 | hsa-miR-3939 | down | -1.1771 | *1* |
| 465 | hsa-miR-31-5p | down | -1.4435 | *1* |
| 466 | hsa-miR-10a-3p | down | -1.4965 | *1* |
| 467 | hsa-miR-548aj-5p | down | -1.4965 | *1* |
| 468 | hsa-miR-4419a | down | -1.4803 | *1* |
| 469 | hsa-miR-6791-5p | down | -1.5328 | *1* |
| 470 | hsa-miR-3190-5p | down | -1.4803 | *1* |
| 471 | hsa-miR-1825 | down | -1.4803 | *1* |
| 472 | hsa-miR-3173-3p | up | 1.0562 | *1* |
| 473 | hsa-miR-376b-3p | down | -2.067 | *1* |
| 474 | hsa-miR-6781-5p | up | 1.0562 | *1* |
| 475 | hsa-miR-1827 | down | -1.4803 | *1* |
| 476 | hsa-miR-7110-3p | down | -1.0424 | *1* |
| 477 | hsa-miR-548g-5p | down | -1.4965 | *1* |
| 478 | hsa-miR-548ap-3p | down | -2.067 | *1* |
| 479 | hsa-miR-516a-5p | down | -2.067 | *1* |
| 480 | hsa-miR-3148 | down | -2.067 | *1* |
| 481 | hsa-miR-4458 | down | -1.4803 | *1* |
| 482 | hsa-miR-7113-5p | down | -2.067 | *1* |
| 483 | hsa-miR-887-5p | down | -1.4803 | *1* |
| 484 | hsa-miR-6777-3p | down | -1.4803 | *1* |
| 485 | hsa-miR-6718-5p | down | -1.5993 | *1* |
| 486 | hsa-miR-26a-1-3p | down | -2.067 | *1* |
| 487 | hsa-miR-3677-3p | down | -1.2177 | *1* |
| 488 | hsa-miR-6855-3p | down | -1.068 | *1* |
| 489 | hsa-miR-4799-5p | down | -1.4803 | *1* |
| 490 | hsa-miR-539-5p | down | -1.4965 | *1* |
| 491 | hsa-miR-3688-3p | down | -1.2017 | *1* |
| 492 | hsa-miR-4479 | down | -1.2251 | *1* |
| 493 | hsa-miR-6736-3p | down | -1.5993 | *1* |
| 494 | hsa-miR-6785-5p | up | 1.1347 | *1* |
| 495 | hsa-miR-200a-5p | down | -1.4552 | *1* |
| 496 | hsa-miR-3682-3p | down | -1.2117 | *1* |
| 497 | hsa-miR-4483 | down | -1.4803 | *1* |
| 498 | hsa-miR-18b-5p | down | -1.4803 | *1* |
| 499 | hsa-miR-4639-5p | down | -1.4803 | *1* |
| 500 | hsa-miR-4510 | down | -1.2702 | *1* |
| 501 | hsa-miR-1234-3p | down | -1.4347 | *1* |
| 502 | hsa-miR-6751-5p | down | -1.8787 | *1* |
| 503 | hsa-miR-3174 | down | -2.067 | *1* |
| 504 | hsa-miR-6848-5p | down | -2.067 | *1* |
| 505 | hsa-miR-4779 | down | -1.4803 | *1* |
| 506 | hsa-miR-3675-5p | down | -1.5607 | *1* |
| 507 | hsa-miR-548aq-3p | down | -1.4965 | *1* |
| 508 | hsa-miR-4318 | down | -1.541 | *1* |
| 509 | hsa-miR-6783-5p | down | -1.4347 | *1* |
| 510 | hsa-miR-576-5p | down | -1.1012 | *1* |
| 511 | hsa-miR-7-1-3p | down | -1.8903 | *1* |
| 512 | hsa-miR-376c-3p | down | -1.4803 | *1* |
| 513 | hsa-miR-135b-5p | down | -1.5993 | *1* |

**Table S5** Mean Ct values for detecting 6 significantly dysregulated exosomal miRNAs in 18 mTLE-HS controls and 18 mTLE+HS patients (namely **Figure 2**).

|  | Ct miR-16-5p | Ct miR-129-5p | ΔCt | Ct miR-16-5p | Ct miR-214-3p | ΔCt | Ct miR-16-5p | Ct miR-219a-5p | ΔCt | Ct miR-16-5p | Ct miR-34c-5p | ΔCt |
| --- | --- | --- | --- | --- | --- | --- | --- | --- | --- | --- | --- | --- |
| TLE-HS 1 | 18.33 | 22.36 | 4.03 | 15.45 | 20.12 | 4.67 | 17.58 | 22.2 | 4.62 | 17.19 | 22.06 | 4.87 |
| TLE-HS 2 | 18 | 22.29 | 4.29 | 15.28 | 20.32 | 5.04 | 19.89 | 24.67 | 4.78 | 18.82 | 23.85 | 5.03 |
| TLE-HS 3 | 16.8 | 21.72 | 4.92 | 14.99 | 20.18 | 5.19 | 17.23 | 22.3 | 5.07 | 18.29 | 23.4 | 5.11 |
| TLE-HS 4 | 18.16 | 23.09 | 4.93 | 17.87 | 23.18 | 5.31 | 18.86 | 23.97 | 5.11 | 17.75 | 22.93 | 5.18 |
| TLE-HS 5 | 17.43 | 22.54 | 5.11 | 16.01 | 21.73 | 5.72 | 16.42 | 21.75 | 5.33 | 16.19 | 21.97 | 5.78 |
| TLE-HS 6 | 18.62 | 23.96 | 5.34 | 15.05 | 20.86 | 5.81 | 17.61 | 22.95 | 5.34 | 18.92 | 24.74 | 5.82 |
| TLE-HS 7 | 18.11 | 23.95 | 5.84 | 16.27 | 22.08 | 5.81 | 15.71 | 21.09 | 5.38 | 18.17 | 24.15 | 5.98 |
| TLE-HS 8 | 17.77 | 23.79 | 6.02 | 17.19 | 23.08 | 5.89 | 16.67 | 22.05 | 5.38 | 18.74 | 24.76 | 6.02 |
| TLE-HS 9 | 17.82 | 23.94 | 6.12 | 15.51 | 21.6 | 6.09 | 16.24 | 21.65 | 5.41 | 16.44 | 22.56 | 6.12 |
| TLE-HS 10 | 16.07 | 22.44 | 6.37 | 16.38 | 22.49 | 6.11 | 13.88 | 19.76 | 5.88 | 17.25 | 23.48 | 6.23 |
| TLE-HS 11 | 17.32 | 23.7 | 6.38 | 16.63 | 22.8 | 6.17 | 15.56 | 21.67 | 6.11 | 17.65 | 24.04 | 6.39 |
| TLE-HS 12 | 18.21 | 24.69 | 6.48 | 18.54 | 24.75 | 6.21 | 14.44 | 20.7 | 6.26 | 18.26 | 24.69 | 6.43 |
| TLE-HS 13 | 18.76 | 25.6 | 6.84 | 16.4 | 22.62 | 6.22 | 17.03 | 23.46 | 6.43 | 16.98 | 23.91 | 6.93 |
| TLE-HS 14 | 17.21 | 24.15 | 6.94 | 16.26 | 22.49 | 6.23 | 17.16 | 23.65 | 6.49 | 18.23 | 25.16 | 6.93 |
| TLE-HS 15 | 18.58 | 25.91 | 7.33 | 16.75 | 23.04 | 6.29 | 16.71 | 23.22 | 6.51 | 19.56 | 26.65 | 7.09 |
| TLE-HS 16 | 16.4 | 24.22 | 7.82 | 17.63 | 24.08 | 6.45 | 16.8 | 24.1 | 7.3 | 19.26 | 26.49 | 7.23 |
| TLE-HS 17 | 18.67 | 26.52 | 7.85 | 18.75 | 25.66 | 6.91 | 15.79 | 23.46 | 7.67 | 16.16 | 23.45 | 7.29 |
| TLE-HS 18 | 17.16 | 25.15 | 7.99 | 16.39 | 23.91 | 7.52 | 15.87 | 23.74 | 7.87 | 18.65 | 25.95 | 7.3 |
| TLE+HS 1 | 19.22 | 23.15 | 3.93 | 18.47 | 22.41 | 3.94 | 17.68 | 20.96 | 3.28 | 17.93 | 22 | 4.07 |
| TLE+HS 2 | 16.77 | 21.1 | 4.33 | 18.23 | 22.34 | 4.11 | 21.53 | 25.14 | 3.61 | 16.6 | 21.31 | 4.71 |
| TLE+HS 3 | 17.98 | 22.77 | 4.79 | 19.36 | 23.52 | 4.16 | 21.61 | 25.38 | 3.77 | 15.95 | 20.67 | 4.72 |
| TLE+HS 4 | 20.31 | 25.14 | 4.83 | 18.29 | 22.52 | 4.23 | 20.69 | 24.5 | 3.81 | 16.94 | 21.86 | 4.92 |
| TLE+HS 5 | 17.33 | 22.17 | 4.84 | 17.08 | 21.32 | 4.24 | 16.63 | 20.51 | 3.88 | 16.36 | 21.29 | 4.93 |
| TLE+HS 6 | 14.61 | 19.5 | 4.89 | 16.29 | 20.71 | 4.42 | 17.09 | 22.17 | 5.08 | 14.83 | 19.85 | 5.02 |
| TLE+HS 7 | 16.08 | 21.04 | 4.96 | 17.48 | 21.91 | 4.43 | 13.28 | 18.36 | 5.08 | 15.35 | 20.52 | 5.17 |
| TLE+HS 8 | 17.51 | 22.53 | 5.02 | 18.45 | 23.08 | 4.63 | 17.41 | 22.54 | 5.13 | 19.23 | 24.46 | 5.23 |
| TLE+HS 9 | 14.8 | 19.82 | 5.02 | 17.47 | 22.2 | 4.73 | 15.75 | 20.98 | 5.23 | 18.29 | 23.56 | 5.27 |
| TLE+HS 10 | 17.75 | 22.79 | 5.04 | 16.67 | 21.41 | 4.74 | 13.92 | 19.23 | 5.31 | 16.59 | 21.88 | 5.29 |
| TLE+HS 11 | 14.8 | 19.86 | 5.06 | 17.15 | 21.93 | 4.78 | 18.58 | 23.96 | 5.38 | 15.5 | 20.88 | 5.38 |
| TLE+HS 12 | 18.36 | 23.44 | 5.08 | 18.11 | 22.95 | 4.84 | 17.37 | 22.95 | 5.58 | 15.98 | 21.66 | 5.68 |
| TLE+HS 13 | 20.32 | 25.65 | 5.33 | 15.77 | 20.91 | 5.14 | 16.36 | 22.15 | 5.79 | 16.39 | 22.18 | 5.79 |
| TLE+HS 14 | 19.16 | 24.61 | 5.45 | 17.06 | 22.3 | 5.24 | 17.61 | 23.43 | 5.82 | 17.08 | 22.98 | 5.9 |
| TLE+HS 15 | 18.19 | 24.23 | 6.04 | 18.45 | 24.17 | 5.72 | 10.93 | 16.81 | 5.88 | 18.95 | 24.97 | 6.02 |
| TLE+HS 16 | 16.58 | 22.76 | 6.18 | 18.67 | 24.46 | 5.79 | 18.33 | 24.23 | 5.9 | 18.03 | 24.31 | 6.28 |
| TLE+HS 17 | 15.79 | 22.33 | 6.54 | 18.61 | 24.54 | 5.93 | 16.5 | 22.4 | 5.9 | 17.89 | 24.27 | 6.38 |
| TLE+HS 18 | 17.77 | 24.45 | 6.68 | 17.39 | 23.42 | 6.03 | 16.31 | 22.79 | 6.48 | 17.77 | 24.35 | 6.58 |
| Continued |  |  |  |  |  |  |  |  |  |  |  |  |
|  | Ct miR-16-5p | Ct miR-421 | ΔCt | Ct miR-16-5p | Ct miR-184 | ΔCt |  |  |  |  |  |  |
| TLE-HS 1 | 18.39 | 22.41 | 4.02 | 16.05 | 20.4 | 4.35 |  |  |  |  |  |  |
| TLE-HS 2 | 18.62 | 22.94 | 4.32 | 18.17 | 22.58 | 4.41 |  |  |  |  |  |  |
| TLE-HS 3 | 17.65 | 22 | 4.35 | 18.04 | 22.5 | 4.46 |  |  |  |  |  |  |
| TLE-HS 4 | 18.68 | 23.14 | 4.46 | 17.46 | 22.24 | 4.78 |  |  |  |  |  |  |
| TLE-HS 5 | 15.6 | 20.45 | 4.85 | 16.65 | 21.48 | 4.83 |  |  |  |  |  |  |
| TLE-HS 6 | 17.58 | 22.47 | 4.89 | 16.09 | 20.98 | 4.89 |  |  |  |  |  |  |
| TLE-HS 7 | 18.47 | 23.41 | 4.94 | 17.37 | 22.3 | 4.93 |  |  |  |  |  |  |
| TLE-HS 8 | 18.12 | 23.07 | 4.95 | 17.55 | 22.69 | 5.14 |  |  |  |  |  |  |
| TLE-HS 9 | 15.73 | 20.7 | 4.97 | 17.75 | 23.27 | 5.52 |  |  |  |  |  |  |
| TLE-HS 10 | 17.13 | 22.21 | 5.08 | 17.99 | 23.76 | 5.77 |  |  |  |  |  |  |
| TLE-HS 11 | 16.39 | 21.58 | 5.19 | 15.77 | 21.63 | 5.86 |  |  |  |  |  |  |
| TLE-HS 12 | 16.77 | 22 | 5.23 | 16.2 | 22.06 | 5.86 |  |  |  |  |  |  |
| TLE-HS 13 | 16.56 | 21.9 | 5.34 | 17.18 | 23.05 | 5.87 |  |  |  |  |  |  |
| TLE-HS 14 | 18.36 | 23.75 | 5.39 | 17.74 | 23.85 | 6.11 |  |  |  |  |  |  |
| TLE-HS 15 | 17.52 | 23.08 | 5.56 | 17.19 | 23.38 | 6.19 |  |  |  |  |  |  |
| TLE-HS 16 | 16.78 | 22.53 | 5.75 | 16.66 | 23.16 | 6.5 |  |  |  |  |  |  |
| TLE-HS 17 | 16.61 | 22.76 | 6.15 | 17.1 | 23.63 | 6.53 |  |  |  |  |  |  |
| TLE-HS 18 | 19.13 | 25.3 | 6.17 | 17.77 | 24.33 | 6.56 |  |  |  |  |  |  |
| TLE+HS 1 | 15.86 | 20.85 | 4.99 | 15.85 | 21.44 | 5.59 |  |  |  |  |  |  |
| TLE+HS 2 | 19.17 | 24.21 | 5.04 | 16.18 | 21.93 | 5.75 |  |  |  |  |  |  |
| TLE+HS 3 | 15.32 | 20.37 | 5.05 | 16.15 | 22.49 | 6.34 |  |  |  |  |  |  |
| TLE+HS 4 | 13.25 | 18.57 | 5.32 | 14.39 | 20.8 | 6.41 |  |  |  |  |  |  |
| TLE+HS 5 | 14.05 | 19.83 | 5.78 | 20.65 | 27.2 | 6.55 |  |  |  |  |  |  |
| TLE+HS 6 | 16.17 | 22.15 | 5.98 | 21.01 | 27.85 | 6.84 |  |  |  |  |  |  |
| TLE+HS 7 | 17.95 | 23.93 | 5.98 | 19.84 | 26.76 | 6.92 |  |  |  |  |  |  |
| TLE+HS 8 | 21.64 | 27.66 | 6.02 | 19.46 | 26.41 | 6.95 |  |  |  |  |  |  |
| TLE+HS 9 | 18.61 | 24.84 | 6.23 | 19.42 | 26.51 | 7.09 |  |  |  |  |  |  |
| TLE+HS 10 | 14.54 | 21.29 | 6.75 | 18.97 | 26.44 | 7.47 |  |  |  |  |  |  |
| TLE+HS 11 | 19.47 | 26.64 | 7.17 | 20.41 | 27.88 | 7.47 |  |  |  |  |  |  |
| TLE+HS 12 | 15.08 | 22.37 | 7.29 | 18.15 | 25.81 | 7.66 |  |  |  |  |  |  |
| TLE+HS 13 | 15.09 | 22.41 | 7.32 | 17.95 | 25.67 | 7.72 |  |  |  |  |  |  |
| TLE+HS 14 | 20.6 | 27.99 | 7.39 | 17.85 | 25.74 | 7.89 |  |  |  |  |  |  |
| TLE+HS 15 | 14.98 | 22.42 | 7.44 | 17.68 | 25.99 | 8.31 |  |  |  |  |  |  |
| TLE+HS 16 | 16.61 | 24.06 | 7.45 | 18 | 26.35 | 8.35 |  |  |  |  |  |  |
| TLE+HS 17 | 18.9 | 26.43 | 7.53 | 15.27 | 24.65 | 9.38 |  |  |  |  |  |  |
| TLE+HS 18 | 18.4 | 26.15 | 7.75 | 16.85 | 26.98 | 10.13 |  |  |  |  |  |  |

**Table S6** The correlation of clinical and pathological variables of 18 mTLE+HS patients with hsa-miR-184 level in plasma exosomal samples.

| Clinical and pathological features | High | Low | *p* value |
| --- | --- | --- | --- |
|  | hsa-miR-184 | hsa-miR-184 |  |
|  | (n=9) | (n=9) |  |
| Gender |  |  | *0.314* |
| Male | 4 | 2 |  |
| Female | 5 | 7 |  |
| Age at onset (years) |  |  | *0.153* |
| <12 | 6 | 3 |  |
| >=12 | 3 | 6 |  |
| Auras |  |  | *0.637* |
| Absent | 5 | 4 |  |
| Present | 4 | 5 |  |
| SE |  |  | *0.229* |
| Absent | 8 | 9 |  |
| Present | 1 | 0 |  |
| IPI |  |  | *0.628* |
| Absent | 5 | 6 |  |
| Present | 4 | 3 |  |
| Frequency (CPS per month) |  |  | ***0.053*** |
| <5 | 6 | 2 |  |
| >=5 | 3 | 7 |  |
| Duration (years) |  |  | *0.628* |
| <16 | 3 | 4 |  |
| >=16 | 6 | 5 |  |
| Pre-operative GTCS |  |  | *0.598* |
| Absent | 2 | 3 |  |
| Present | 7 | 6 |  |
| Hippocampus |  |  | *0.105* |
| Left | 5 | 8 |  |
| Right | 4 | 1 |  |
| FCD |  |  | *1.000* |
| I | 5 | 5 |  |
| II/III | 0/4 | 1/3 |  |

Abbreviations: SE, status epilepticus; IPI, initial precipitating incident; CPS, complex partial seizure; GTCS, generalized tonic clonic seizure; FCD, [focal cortical dysplasia.](https://www.ncbi.nlm.nih.gov/pubmed/27750396)

**Table S7** The detailed information of target genes predicted by all four software (TargetScan, miRDB, miRanda and CLIP-seq).

| miR-129-5p | miR-214-3p | miR-219a-5p | miR-34c-5p | miR-421 | miR-184 |
| --- | --- | --- | --- | --- | --- |
| (n = 324) | (n = 148) | (n = 41) | (n = 64) | (n = 196) | (n = 14) |
| SPIRE1 | MFSD6 | ELOVL7 | ZNF551 | XRN1 | PPP6R1 |
| ZFP106 | GALNT7 | PHKA1 | AP1S2 | POLR2D | CCDC77 |
| SACM1L | HDGF | INSIG1 | ARHGAP26 | ASPH | KIAA0247 |
| BACH2 | PELI3 | CACUL1 | SLC4A7 | TUBB2A | NCOR2 |
| VPS26A | CELF1 | RECK | MET | TRAPPC8 | SF1 |
| NIPBL | GALNTL4 | SLC31A1 | GREM2 | MAT2B | AGRN |
| C16orf80 | CBL | DNAL1 | PPP2R5A | TRAF6 | PPAP2B |
| UBE2O | CTSS | AKAP13 | STK38L | MTA1 | ZNF740 |
| STOX2 | SNX18 | THRB | TPD52 | PLCXD3 | LRRC8A |
| ZNF609 | ERC1 | ZCCHC14 | VAMP2 | PLEKHA5 | SLC6A9 |
| ETV6 | TMEM161B | LPP | SATB1 | ANKRD17 | GNB1 |
| CCDC43 | PFKFB4 | FBXO3 | SEMA4C | BMF | C20orf112 |
| AUTS2 | CD276 | LURAP1L | PTPRD | TESK2 | KIAA0664 |
| ROCK1 | NAGPA | ASH1L | TP53INP2 | C6orf106 | EIF2C2 |
| STAT5B | ACLY | MLL2 | EMC1 | ARHGAP12 |  |
| IFT80 | C16orf72 | DAZAP1 | MSL2 | SUPT16H |  |
| ACTN1 | HCLS1 | SMC4 | TTC19 | NCKAP1 |  |
| MED26 | PPIL2 | NEK6 | ZFHX4 | JPH1 |  |
| GALNT1 | JUP | DDAH1 | ASXL2 | TXNRD1 |  |
| RNF217 | ABCC5 | KBTBD8 | CWC25 | FAM76B |  |
| UBE2R2 | ZFAND3 | SLC35F5 | FAM207A | GMNN |  |
| FAM38B | TYRO3 | MKL1 | RIMS3 | AKAP1 |  |
| KCNK1 | USP19 | KRR1 | TANC2 | FAM38B |  |
| ZNF678 | FIBCD1 | UBR1 | RGS17 | MPV17 |  |
| SBF2 | SCAMP4 | FMNL2 | PPARGC1B | UTP3 |  |
| AMMECR1L | PPP1R8 | RNF6 | SLC2A13 | RMND5A |  |
| FAM160B1 | ASXL1 | FOXJ3 | NFE2L1 | JAKMIP2 |  |
| PHACTR4 | LAMC3 | SLC41A1 | SEMA4B | VPS29 |  |
| NPEPPS | RAPGEFL1 | LEPR | MTUS1 | ZNF609 |  |
| THUMPD1 | SLC9A3R2 | PKD2 | KITLG | MLLT6 |  |
| NEDD1 | MED19 | ATG14 | PRKAG1 | PCBP2 |  |
| SEL1L | GPSM1 | CRLF3 | NRIP3 | DFFB |  |
| MATR3 | REEP4 | SLC27A6 | PREB | ZNF721 |  |
| LIMS1 | MFSD4 | LAPTM4A | NAV1 | HECA |  |
| HIPK1 | CCNL2 | TGFBR2 | PPP1R10 | PIGA |  |
| PDS5A | GLYCTK | PHF19 | NUP43 | FAM214A |  |
| TMX3 | ATP8B2 | CDH1 | SGPP1 | AFF4 |  |
| FAM45A | NSUN4 | KLF16 | SUCO | MTRR |  |
| PTP4A1 | NUFIP2 | ISL1 | POGZ | DNAJC27 |  |
| FMR1 | PAPPA | GBA2 | RPS6KL1 | PACRGL |  |
| CACNB4 | RPIA | UBE2Z | BAZ2A | HMG20A |  |
| RB1 | NPTX2 |  | ZBTB9 | BCOR |  |
| CAB39 | SNX12 |  | ANKS1A | RBBP7 |  |
| ZNF608 | DOCK9 |  | MARCKS | RHOBTB1 |  |
| EP300 | PPIH |  | ALCAM | YTHDF1 |  |
| MKNK2 | TBC1D10B |  | GAB1 | ARHGAP19 |  |
| TET1 | LEPROTL1 |  | FAM123B | EIF2S1 |  |
| LMNA | CDC42SE1 |  | C7orf49 | IL1RAP |  |
| CADM1 | TWF1 |  | KLHDC3 | TOMM70A |  |
| APBB2 | TMEM258 |  | CNOT4 | ILF2 |  |
| KDM2A | PDLIM5 |  | MARCKSL1 | EPS8 |  |
| AP3B1 | ATXN1L |  | SYT1 | ZNF263 |  |
| MXD4 | FAM133B |  | CSNK1G3 | EPHA7 |  |
| MYT1L | BCL11A |  | SLC2A8 | EDEM3 |  |
| CDC42EP3 | MAP3K4 |  | KIAA1217 | GXYLT2 |  |
| SNRNP27 | EZH1 |  | IRAK2 | NIPBL |  |
| DSE | TATDN2 |  | CNOT6 | ATAD2B |  |
| CNN3 | SMYD5 |  | SIDT2 | RSU1 |  |
| INO80D | CSF1 |  | GPS2 | NR2C2 |  |
| MYLIP | ZNRF2 |  | ZNF304 | RIT1 |  |
| MED14 | SMOC1 |  | PDXK | CASD1 |  |
| FAM13B | C6orf192 |  | DNAJC16 | SNRNP70 |  |
| ZFX | CLASP1 |  | LDHA | CDK12 |  |
| ABHD3 | TRIP10 |  | TMEM55A | CRIM1 |  |
| ATP8B2 | SYS1 |  |  | SENP5 |  |
| EIF2C3 | NUMA1 |  |  | RNMT |  |
| PRPF4 | SLC25A39 |  |  | CD164 |  |
| AP2B1 | BSDC1 |  |  | PCYT1A |  |
| HDGFRP3 | CRKL |  |  | FAR1 |  |
| CGGBP1 | TRAK1 |  |  | ATG2B |  |
| RNF165 | NUCB1 |  |  | SPR |  |
| AHDC1 | DNAJB5 |  |  | WDR35 |  |
| EI24 | ELMSAN1 |  |  | POLR1B |  |
| C11orf54 | FOXJ3 |  |  | PLK2 |  |
| FRYL | TLE3 |  |  | RFX3 |  |
| NR2C2 | MED20 |  |  | SLC4A7 |  |
| MGLL | TTYH3 |  |  | CYTH3 |  |
| UBE2K | GJA1 |  |  | CLDN11 |  |
| FHL1 | PLAGL2 |  |  | ZNF675 |  |
| DAB2IP | FAM134A |  |  | FNBP1L |  |
| SNAPC1 | GHITM |  |  | PRCC |  |
| RSBN1 | TBL1XR1 |  |  | GALNT3 |  |
| VEZT | PVRL1 |  |  | PREPL |  |
| IRF2BPL | SEC24C |  |  | FAM120A |  |
| SPRY4 | TGFBRAP1 |  |  | SP3 |  |
| GLCCI1 | RBM34 |  |  | MGEA5 |  |
| SNX3 | PPM1H |  |  | FCHSD2 |  |
| CTNND1 | PISD |  |  | PLDN |  |
| RASSF5 | PRKAR1A |  |  | S100A2 |  |
| ACSL4 | PAFAH1B1 |  |  | DLX3 |  |
| CTDSPL2 | ACSL1 |  |  | EDARADD |  |
| THSD4 | RNF169 |  |  | ZNF148 |  |
| RAI14 | USP3 |  |  | CHD2 |  |
| TCOF1 | MTMR12 |  |  | C16orf52 |  |
| NR4A2 | CNIH |  |  | CTDSPL2 |  |
| C5orf28 | FAM178A |  |  | STYK1 |  |
| C14orf28 | TBC1D24 |  |  | FAM32A |  |
| TOX | NEK6 |  |  | FUT10 |  |
| CD2AP | TRIM59 |  |  | PTBP3 |  |
| ZBTB20 | ATP2B4 |  |  | MLLT4 |  |
| LRIG3 | CDIPT |  |  | GAN |  |
| PRDM1 | ABHD2 |  |  | SPRED2 |  |
| BTBD3 | FGFR1 |  |  | ELAVL2 |  |
| ARHGEF10L | CRTC3 |  |  | TOLLIP |  |
| KAT6B | INO80C |  |  | WIPI2 |  |
| SHISA9 | LPCAT1 |  |  | SPATS2L |  |
| TBC1D15 | KCNC4 |  |  | ZC3H12C |  |
| KLHL24 | HTT |  |  | ELL2 |  |
| SLC7A1 | AHDC1 |  |  | EFR3A |  |
| GDE1 | CELSR2 |  |  | TMEM259 |  |
| SMG1 | CPSF4 |  |  | EYA1 |  |
| ATXN7 | HSPA12A |  |  | TNKS2 |  |
| ARL5A | AHCYL2 |  |  | MTF1 |  |
| CAMK4 | COPS7B |  |  | DCAF17 |  |
| SESN3 | PSMD10 |  |  | ARID4B |  |
| ATP5L | SLC12A6 |  |  | CORO2A |  |
| CHMP2B | RFX7 |  |  | FAM126B |  |
| TMTC4 | DBNDD2 |  |  | MTOR |  |
| PAQR5 | PTEN |  |  | AASDHPPT |  |
| AMMECR1 | COL4A5 |  |  | PTPRB |  |
| RRAGD | VEZT |  |  | CAPZA1 |  |
| SESTD1 | NDUFAF4 |  |  | SPAST |  |
| SH3BGRL | VAV2 |  |  | NR3C1 |  |
| NUP43 | P4HTM |  |  | SV2A |  |
| ZDHHC23 | FAM20B |  |  | MYH10 |  |
| HNRNPK | KPNA1 |  |  | SMARCD1 |  |
| FKBP5 | KIAA0226 |  |  | SLC25A24 |  |
| C3orf38 | AHNAK2 |  |  | KLF6 |  |
| SOS2 | MYO18A |  |  | KIAA2026 |  |
| RAB11FIP5 | ZNF395 |  |  | NR1D2 |  |
| GREM2 | ACER3 |  |  | SGPP1 |  |
| DFFA | MAFG |  |  | EIF2S2 |  |
| SBK1 | ANKRD6 |  |  | MAML1 |  |
| SSH2 | MYO1D |  |  | TSN |  |
| GAPVD1 | GAN |  |  | RTTN |  |
| HNRNPA3 | KIAA0247 |  |  | PLS3 |  |
| CDK6 | RPS6KL1 |  |  | QKI |  |
| RNF182 | SNX11 |  |  | PSMD10 |  |
| MEIS2 | GPATCH1 |  |  | RSBN1L |  |
| TERF2 | CKAP5 |  |  | APPL1 |  |
| PPP2CB | MPDU1 |  |  | POLQ |  |
| TRIO | RASSF5 |  |  | MSL2 |  |
| VASH2 | SESN1 |  |  | CALU |  |
| FIGN | TMEM33 |  |  | SPRY1 |  |
| MAMDC2 | PNPLA6 |  |  | CCDC14 |  |
| PCDH7 | HNRNPU |  |  | KDM5A |  |
| SC5DL | NUAK2 |  |  | HOXA9 |  |
| FBXW7 | HS2ST1 |  |  | ZFP36L2 |  |
| ADNP2 |  |  |  | PNPO |  |
| DUSP10 |  |  |  | SUV420H1 |  |
| PPP1R3B |  |  |  | NDFIP1 |  |
| IFFO2 |  |  |  | PRUNE2 |  |
| MLL3 |  |  |  | NRAS |  |
| USP9X |  |  |  | FCF1 |  |
| TAOK1 |  |  |  | ONECUT2 |  |
| ZFP36L1 |  |  |  | C3orf58 |  |
| PPP1R12A |  |  |  | IPO5 |  |
| ADD3 |  |  |  | BNIP3L |  |
| ITGA6 |  |  |  | SUZ12 |  |
| NR3C2 |  |  |  | JARID2 |  |
| MRPL44 |  |  |  | CACNB4 |  |
| FAM208B |  |  |  | RDX |  |
| UNK |  |  |  | CUL4B |  |
| USP6NL |  |  |  | TBC1D4 |  |
| CAMTA1 |  |  |  | NMU |  |
| ENOSF1 |  |  |  | UBE2B |  |
| U2SURP |  |  |  | CASP3 |  |
| ZMAT2 |  |  |  | EPHA4 |  |
| EIF4G3 |  |  |  | FAM8A1 |  |
| SPOCK3 |  |  |  | LUC7L2 |  |
| ING3 |  |  |  | CNOT6L |  |
| HDAC9 |  |  |  | TROVE2 |  |
| TGIF2 |  |  |  | MBD2 |  |
| CDYL |  |  |  | HMGB3 |  |
| SLBP |  |  |  | TMEM181 |  |
| ERAP1 |  |  |  | TMPO |  |
| ELK4 |  |  |  | HOOK1 |  |
| RPS6KA5 |  |  |  | ATP6V1C1 |  |
| NCKAP5 |  |  |  | PRRC1 |  |
| FANCI |  |  |  | TMEM65 |  |
| ZBTB4 |  |  |  | ACBD3 |  |
| KPNA6 |  |  |  | MGAT4A |  |
| CDKN1C |  |  |  | TBC1D30 |  |
| RFX7 |  |  |  | NFYA |  |
| APC |  |  |  | PAPOLG |  |
| MAP1LC3B |  |  |  | UTS2D |  |
| RBMS1 |  |  |  | ASAP1 |  |
| EBF1 |  |  |  | RHBDF1 |  |
| ERI2 |  |  |  | FAM160B1 |  |
| PDPK1 |  |  |  | MEIS2 |  |
| PBX3 |  |  |  | PRKAA2 |  |
| AMOTL2 |  |  |  | STRN3 |  |
| FOXP2 |  |  |  | RASA1 |  |
| AS3MT |  |  |  | DDX60L |  |
| FBXO33 |  |  |  | PPP1CC |  |
| SLC25A25 |  |  |  | SREK1IP1 |  |
| C6orf120 |  |  |  |  |  |
| PDCD6 |  |  |  |  |  |
| GLYR1 |  |  |  |  |  |
| ZBTB10 |  |  |  |  |  |
| SUN2 |  |  |  |  |  |
| HS6ST2 |  |  |  |  |  |
| KLHL5 |  |  |  |  |  |
| FAM63B |  |  |  |  |  |
| SLU7 |  |  |  |  |  |
| SLC39A14 |  |  |  |  |  |
| JPH3 |  |  |  |  |  |
| EGFR |  |  |  |  |  |
| PAIP1 |  |  |  |  |  |
| EEA1 |  |  |  |  |  |
| HMGB1 |  |  |  |  |  |
| POLR2E |  |  |  |  |  |
| MAP3K13 |  |  |  |  |  |
| CXorf23 |  |  |  |  |  |
| ADARB1 |  |  |  |  |  |
| OXR1 |  |  |  |  |  |
| VCP |  |  |  |  |  |
| STEAP2 |  |  |  |  |  |
| NWD1 |  |  |  |  |  |
| KCTD1 |  |  |  |  |  |
| MEX3A |  |  |  |  |  |
| PITPNB |  |  |  |  |  |
| MAPRE1 |  |  |  |  |  |
| MIER1 |  |  |  |  |  |
| AMPH |  |  |  |  |  |
| YWHAB |  |  |  |  |  |
| MARCKSL1 |  |  |  |  |  |
| CALM1 |  |  |  |  |  |
| PHF8 |  |  |  |  |  |
| RPS6KB1 |  |  |  |  |  |
| FNIP2 |  |  |  |  |  |
| NFX1 |  |  |  |  |  |
| ERBB2IP |  |  |  |  |  |
| YIPF5 |  |  |  |  |  |
| DAG1 |  |  |  |  |  |
| UQCRB |  |  |  |  |  |
| SH3KBP1 |  |  |  |  |  |
| EPHB1 |  |  |  |  |  |
| UBASH3B |  |  |  |  |  |
| RBPMS |  |  |  |  |  |
| SERP1 |  |  |  |  |  |
| ITPR1 |  |  |  |  |  |
| PAPD5 |  |  |  |  |  |
| GRIK2 |  |  |  |  |  |
| AADAT |  |  |  |  |  |
| NUP50 |  |  |  |  |  |
| LSM12 |  |  |  |  |  |
| BCL10 |  |  |  |  |  |
| CELF1 |  |  |  |  |  |
| TBL1XR1 |  |  |  |  |  |
| MEF2A |  |  |  |  |  |
| ETS1 |  |  |  |  |  |
| ETV1 |  |  |  |  |  |
| NXT2 |  |  |  |  |  |
| RAB33B |  |  |  |  |  |
| PRKCB |  |  |  |  |  |
| LBR |  |  |  |  |  |
| GALNT12 |  |  |  |  |  |
| RUNX1T1 |  |  |  |  |  |
| BPTF |  |  |  |  |  |
| ATF5 |  |  |  |  |  |
| SOX2 |  |  |  |  |  |
| PHF12 |  |  |  |  |  |
| TXNDC15 |  |  |  |  |  |
| ATP2B4 |  |  |  |  |  |
| MFAP3L |  |  |  |  |  |
| SAMSN1 |  |  |  |  |  |
| RBMS3 |  |  |  |  |  |
| SOX4 |  |  |  |  |  |
| DTD2 |  |  |  |  |  |
| PMEPA1 |  |  |  |  |  |
| NALCN |  |  |  |  |  |
| ELAVL1 |  |  |  |  |  |
| FAM114A2 |  |  |  |  |  |
| ZIC2 |  |  |  |  |  |
| ALPL |  |  |  |  |  |
| NEBL |  |  |  |  |  |
| MRPL35 |  |  |  |  |  |
| REEP1 |  |  |  |  |  |
| ABCC5 |  |  |  |  |  |
| BMPR2 |  |  |  |  |  |
| SGMS1 |  |  |  |  |  |
| OTUD6B |  |  |  |  |  |
| LANCL1 |  |  |  |  |  |
| PBX1 |  |  |  |  |  |
| CBLB |  |  |  |  |  |
| MYCBP2 |  |  |  |  |  |
| CAMK2N1 |  |  |  |  |  |
| DNAJC13 |  |  |  |  |  |
| ATG14 |  |  |  |  |  |
| PHIP |  |  |  |  |  |
| FLT1 |  |  |  |  |  |
| CBX7 |  |  |  |  |  |
| UNC5D |  |  |  |  |  |
| YWHAH |  |  |  |  |  |
| RBM47 |  |  |  |  |  |
| SDAD1 |  |  |  |  |  |
| CCDC88A |  |  |  |  |  |
| ZNF281 |  |  |  |  |  |
| MAN1A2 |  |  |  |  |  |
| SASH1 |  |  |  |  |  |
| HLCS |  |  |  |  |  |
| PHF17 |  |  |  |  |  |
| WWTR1 |  |  |  |  |  |
| ETV5 |  |  |  |  |  |
| RBM12 |  |  |  |  |  |
| GSPT2 |  |  |  |  |  |
| SEC14L1 |  |  |  |  |  |
| UBE2H |  |  |  |  |  |
| TSHZ1 |  |  |  |  |  |
| SPOP |  |  |  |  |  |
| UBR1 |  |  |  |  |  |
| ASAP1 |  |  |  |  |  |
| SPOCK1 |  |  |  |  |  |
| FOXN3 |  |  |  |  |  |
| SP3 |  |  |  |  |  |
| SIM2 |  |  |  |  |  |
| LRRFIP1 |  |  |  |  |  |
| CBX4 |  |  |  |  |  |
| ATAD2B |  |  |  |  |  |
| NFIB |  |  |  |  |  |
| GINS3 |  |  |  |  |  |
| COX15 |  |  |  |  |  |
| ZEB2 |  |  |  |  |  |

**Table S8** The target genes pool with target score >80 correlated with potential implications in epilepsy using miRDB.

| **miRNA Name** | **Target Score** | **Gene Symbol** | **Gene Description** |
| --- | --- | --- | --- |
| hsa-miR-129-5p | 99 | [CACNG2](http://www.ncbi.nlm.nih.gov/entrez/query.fcgi?db=gene&cmd=Retrieve&dopt=full_report&list_uids=10369) | calcium voltage-gated channel auxiliary subunit gamma 2 |
|  | 99 | [CACNB4](http://www.ncbi.nlm.nih.gov/entrez/query.fcgi?db=gene&cmd=Retrieve&dopt=full_report&list_uids=785) | calcium voltage-gated channel auxiliary subunit beta 4 |
|  | 98 | [SLC17A6](http://www.ncbi.nlm.nih.gov/entrez/query.fcgi?db=gene&cmd=Retrieve&dopt=full_report&list_uids=57084) | solute carrier family 17 member 6 |
|  | 98 | [NRXN1](http://www.ncbi.nlm.nih.gov/entrez/query.fcgi?db=gene&cmd=Retrieve&dopt=full_report&list_uids=9378) | neurexin 1 (Ca[2+]-dependent neurexin/neuroligin complexes at synapses of CNS efficient neurotransmission) |
|  | 97 | [CAMK4](http://www.ncbi.nlm.nih.gov/entrez/query.fcgi?db=gene&cmd=Retrieve&dopt=full_report&list_uids=814) | calcium/calmodulin dependent protein kinase IV |
|  | 96 | [SCN8A](http://www.ncbi.nlm.nih.gov/entrez/query.fcgi?db=gene&cmd=Retrieve&dopt=full_report&list_uids=6334) | sodium voltage-gated channel alpha subunit 8 |
|  | 96 | [GABBR2](http://www.ncbi.nlm.nih.gov/entrez/query.fcgi?db=gene&cmd=Retrieve&dopt=full_report&list_uids=9568) | gamma-aminobutyric acid type B receptor subunit 2 |
|  | 95 | [CAMK2N1](http://www.ncbi.nlm.nih.gov/entrez/query.fcgi?db=gene&cmd=Retrieve&dopt=full_report&list_uids=55450) | calcium/calmodulin dependent protein kinase II inhibitor 1 |
|  | 94 | [NEUROD2](http://www.ncbi.nlm.nih.gov/entrez/query.fcgi?db=gene&cmd=Retrieve&dopt=full_report&list_uids=4761) | neuronal differentiation 2 |
|  | 93 | [KCTD1](http://www.ncbi.nlm.nih.gov/entrez/query.fcgi?db=gene&cmd=Retrieve&dopt=full_report&list_uids=284252) | potassium channel tetramerization domain containing 1 |
|  | 91 | [SLC13A1](http://www.ncbi.nlm.nih.gov/entrez/query.fcgi?db=gene&cmd=Retrieve&dopt=full_report&list_uids=6561) | solute carrier family 13 member 1 |
|  | 90 | [CAMK1D](http://www.ncbi.nlm.nih.gov/entrez/query.fcgi?db=gene&cmd=Retrieve&dopt=full_report&list_uids=57118) | calcium/calmodulin dependent protein kinase ID |
| hsa-miR-214-3p | 95 | [SLC8A1](http://www.ncbi.nlm.nih.gov/entrez/query.fcgi?db=gene&cmd=Retrieve&dopt=full_report&list_uids=6546) | solute carrier family 8 member A1 |
|  | 92 | [SLC23A2](http://www.ncbi.nlm.nih.gov/entrez/query.fcgi?db=gene&cmd=Retrieve&dopt=full_report&list_uids=9962) | solute carrier family 23 member 2 |
|  | 91 | [KCNC2](http://www.ncbi.nlm.nih.gov/entrez/query.fcgi?db=gene&cmd=Retrieve&dopt=full_report&list_uids=3747) | potassium voltage-gated channel subfamily C member 2 |
|  | 91 | [SLC25A39](http://www.ncbi.nlm.nih.gov/entrez/query.fcgi?db=gene&cmd=Retrieve&dopt=full_report&list_uids=51629) | solute carrier family 25 member 39 |
| hsa-miR-219a-5p | 98 | [SLC16A7](http://www.ncbi.nlm.nih.gov/entrez/query.fcgi?db=gene&cmd=Retrieve&dopt=full_report&list_uids=9194) | solute carrier family 16 member 7 |
|  | 96 | [KCNA4](http://www.ncbi.nlm.nih.gov/entrez/query.fcgi?db=gene&cmd=Retrieve&dopt=full_report&list_uids=3739) | potassium voltage-gated channel subfamily A member 4 |
|  | 92 | [SLC35E1](http://www.ncbi.nlm.nih.gov/entrez/query.fcgi?db=gene&cmd=Retrieve&dopt=full_report&list_uids=79939) | solute carrier family 35 member E1 |
|  | 91 | [SLC46A3](http://www.ncbi.nlm.nih.gov/entrez/query.fcgi?db=gene&cmd=Retrieve&dopt=full_report&list_uids=283537) | solute carrier family 46 member 3 |
| hsa-miR-34c-5p | 98 | [SCN2B](http://www.ncbi.nlm.nih.gov/entrez/query.fcgi?db=gene&cmd=Retrieve&dopt=full_report&list_uids=6327) | sodium voltage-gated channel beta subunit 2 |
|  | 96 | [VAMP2](http://www.ncbi.nlm.nih.gov/entrez/query.fcgi?db=gene&cmd=Retrieve&dopt=full_report&list_uids=6844) | vesicle associated membrane protein 2 (synaptobrevin 2) |
|  | 96 | [CACNA1E](http://www.ncbi.nlm.nih.gov/entrez/query.fcgi?db=gene&cmd=Retrieve&dopt=full_report&list_uids=777) | calcium voltage-gated channel subunit alpha1 E |
|  | 95 | [SLC25A27](http://www.ncbi.nlm.nih.gov/entrez/query.fcgi?db=gene&cmd=Retrieve&dopt=full_report&list_uids=9481) | solute carrier family 25 member 27 |
|  | 93 | [GABRA3](http://www.ncbi.nlm.nih.gov/entrez/query.fcgi?db=gene&cmd=Retrieve&dopt=full_report&list_uids=2556) | gamma-aminobutyric acid type A receptor alpha3 subunit |
|  | 90 | [SLC4A7](http://www.ncbi.nlm.nih.gov/entrez/query.fcgi?db=gene&cmd=Retrieve&dopt=full_report&list_uids=9497) | solute carrier family 4 member 7 |
| hsa-miR-421 | 95 | [SYT14](http://www.ncbi.nlm.nih.gov/entrez/query.fcgi?db=gene&cmd=Retrieve&dopt=full_report&list_uids=255928) | synaptotagmin 14 |
| hsa-miR-184 | 85 | [TNPO2](http://www.ncbi.nlm.nih.gov/entrez/query.fcgi?db=gene&cmd=Retrieve&dopt=full_report&list_uids=30000) | transportin 2 |
|  | 80 | [CAMK2B](http://www.ncbi.nlm.nih.gov/entrez/query.fcgi?db=gene&cmd=Retrieve&dopt=full_report&list_uids=816) | calcium/calmodulin dependent protein kinase II beta |

**Table S9** The top ranked pathways generated through input 6 differentially expressed miRNAs submitted to DIANA mirPath.

| KEGG pathway | p-value | regulated genes | miRNAs involved |
| --- | --- | --- | --- |
| Fatty acid biosynthesis | *1.92E-16* | 2 | 2 |
| Proteoglycans in cancer | *3.75E-08* | 46 | 5 |
| Glioma | *2.70E-05* | 18 | 4 |
| Arrhythmogenic right ventricular cardiomyopathy (ARVC) | *2.70E-05* | 12 | 4 |
| **Hippo signaling pathway** | *2.70E-05* | 30 | 5 |
| Chronic myeloid leukemia | *6.33E-05* | 20 | 4 |
| Biotin metabolism | *0.000107164* | 1 | 1 |
| Gap junction | *0.000141301* | 19 | 4 |
| Regulation of actin cytoskeleton | *0.000141301* | 41 | 4 |
| Prostate cancer | *0.00014732* | 25 | 4 |
| Fatty acid metabolism | *0.000274556* | 7 | 4 |
| **p53 signaling pathway** | *0.000274556* | 20 | 4 |
| Pathways in cancer | *0.000332027* | 64 | 5 |
| Melanoma | *0.000443462* | 17 | 4 |
| Bacterial invasion of epithelial cells | *0.000562641* | 17 | 4 |
| ECM-receptor interaction | *0.000737465* | 13 | 5 |
| Viral carcinogenesis | *0.001123295* | 30 | 5 |
| Adherens junction | *0.001971541* | 20 | 4 |
| mRNA surveillance pathway | *0.003518854* | 20 | 5 |
| Shigellosis | *0.004149582* | 16 | 4 |
| Focal adhesion | *0.004949729* | 40 | 5 |
| Central carbon metabolism in cancer | *0.006496297* | 15 | 4 |
| **TGF-beta signaling pathway** | *0.009810612* | 17 | 5 |
| Bladder cancer | *0.011076194* | 12 | 4 |
| **HIF-1 signaling pathway** | *0.011425849* | 22 | 4 |
| Renal cell carcinoma | *0.012048725* | 16 | 4 |
| Acute myeloid leukemia | *0.012048725* | 14 | 4 |
| Cell cycle | *0.013759496* | 26 | 5 |
| Long-term depression | *0.014012767* | 12 | 4 |
| Colorectal cancer | *0.020948096* | 13 | 4 |
| Endometrial cancer | *0.022964901* | 13 | 4 |
| Thyroid cancer | *0.022964901* | 7 | 4 |
| Small cell lung cancer | *0.024014076* | 18 | 5 |
| Hepatitis B | *0.049223908* | 25 | 4 |
| Lysine degradation | *0.049313622* | 9 | 4 |
| Spliceosome | *0.049313622* | 21 | 4 |
| **mTOR signaling pathway** | *0.049313622* | 13 | 4 |
| Dorso-ventral axis formation | *0.049313622* | 8 | 4 |
| Tight junction | *0.049313622* | 26 | 4 |
| Measles | *0.049313622* | 26 | 4 |
| **Neurotrophin signaling pathway** | *0.049313622* | 21 | 5 |

**Table S10** Epilepsy-related important pathways potentially regulated by altered 6 miRNAs in mTLE+HS.

| KEGG | p-value | miRNAs involved in | Regulated specific genes |
| --- | --- | --- | --- |
| pathway |  | pathway regulation |  |
| Neurotrophin | *0.049* | hsa-miR-129-5p | BRAF,GSK3B,SOS2,MAP3K3,CRK,PRKCD,PIK3CA |
| signaling |  | hsa-miR-214-3p | NRAS,CRKL,TP53,RPS6KA3,MAPK1,ATF4 |
| pathway |  | hsa-miR-34c-5p | CALM3,AKT2,ARHGDIG,NFKBIA,ARHGDIA,PTPN11 |
|  |  | hsa-miR-421 | NRAS,MAP2K7,FRS2 |
|  |  | hsa-miR-184 | FRS2,ARHGDIA |
| mTOR | *0.049* | hsa-miR-129-5p | BRAF,RRAGD,PIK3CA,PTEN,RPS6KB1 |
| signaling |  | hsa-miR-214-3p | RPS6KA3,VEGFA,PTEN,MAPK1 |
| pathway |  | hsa-miR-34c-5p | AKT2,ULK1 |
|  |  | hsa-miR-421 | PRKAA1,MTOR,ULK1,RRAGB |
| HIF-1 | *0.01* | hsa-miR-34c-5p | GAPDH,IFNGR2,AKT2,LDHA,HK1,MKNK2,SERPINE1 |
| signaling |  | hsa-miR-129-5p | ARNT,CDKN1B,IGF1R,EP300,PIK3CA,RPS6KB1 |
| pathway |  | hsa-miR-214-3p | STAT3,PFKFB2,ALDOA,VEGFA,MAPK1,PGK1,TIMP1 |
|  |  | hsa-miR-421 | CDKN1B,TFRC,HK1,MTOR,SERPINE1,PGK1 |
| TGF-beta | *0.0098* | hsa-miR-34c-5p | THBS1,E2F5,MYC,PPP2R1A |
| signaling |  | hsa-miR-129-5p | ROCK1,INHBA,ACVR2B,DCN,E2F5,EP300,BMPR2,RPS6KB1 |
| pathway |  | hsa-miR-214-3p | FST,THBS1,MAPK1 |
|  |  | hsa-miR-184 | LTBP1 |
|  |  | hsa-miR-421 | SMAD2,ACVR2B,ZFYVE9,PPPP2CB,LTBP1 |
| p53 | *2.70x10^-4^* | hsa-miR-34c-5p | CDK4,THBS1,CCND2,CDK6,STEAP3,SERPINE1 |
| signaling |  | hsa-miR-214-3p | CCNG1,THBS1,TP53,SHISA5,IGFBP3,PTEN,MDM2 |
| pathway |  | hsa-miR-129-5p | ZMAT3,CCND2,PERP,RRM2B,CASP3,CCND1,PTEN,MDM2 |
|  |  | hsa-miR-421 | ZMAT3,CDK6,PMAIP1,CASP3,SESN3,SERPINE1,MDM2,CCND3 |
| Hippo | *2.70x10^-5^* | hsa-miR-129-5p | GSK3B,YAP1,WNT5A,CCND2,FZD6,CCND1,TEAD1,PARD3,LATS2,BMPR2 |
| signaling |  | hsa-miR-214-3p | YAP1,ACTG1,TEAD1,AJUBA |
| pathway |  | hsa-miR-34c-5p | PPP1CA,ACTB,PPP1CC,CCND2,FZD3,MYC,PPP2R1A,LLGL1,SERPINE1,BIRC2 |
|  |  | hsa-miR-421 | YWHAH,SMAD2,PPP1CC,BIRC5,YWHAB,CTNNA1,PPP2CB,FBXW11,SERPINE1,CCND3 |
|  |  | hsa-miR-184 | YWHAG |

The list was conducted by submitting the list of 6 differentially expressed miRNAs (hsa-miR-129-5p, -214-3p, -219a-5p, -34c-5p, -421 and -184) to the DIANA-mirPath database.
